# Prediction of the Interactions of a Large Number of Per- and Poly-Fluoroalkyl Substances with Ten Nuclear Receptors

**DOI:** 10.1021/acs.est.3c05974

**Published:** 2024-02-29

**Authors:** Ettayapuram Ramaprasad Azhagiya Singam, Kathleen A. Durkin, Michele A. La Merrill, J. David Furlow, Jen-Chywan Wang, Martyn T. Smith

**Affiliations:** † Molecular Graphics and Computation Facility, College of Chemistry, University of California, Berkeley, California 94720, United States; ‡ Department of Environmental Toxicology, University of California, Davis, California 95616, United States; § Department of Neurobiology, Physiology and Behavior, University of California, Davis California 95616, United States; ∥ Department of Nutritional Sciences and Toxicology, University of California, Berkeley, California 94720, United States; ⊥ Division of Environmental Health Sciences, School of Public Health, University of California Berkeley, Berkeley, California 94720, United States

**Keywords:** PFASs, estrogen receptor, androgen receptor PPAR, endocrine disruption

## Abstract

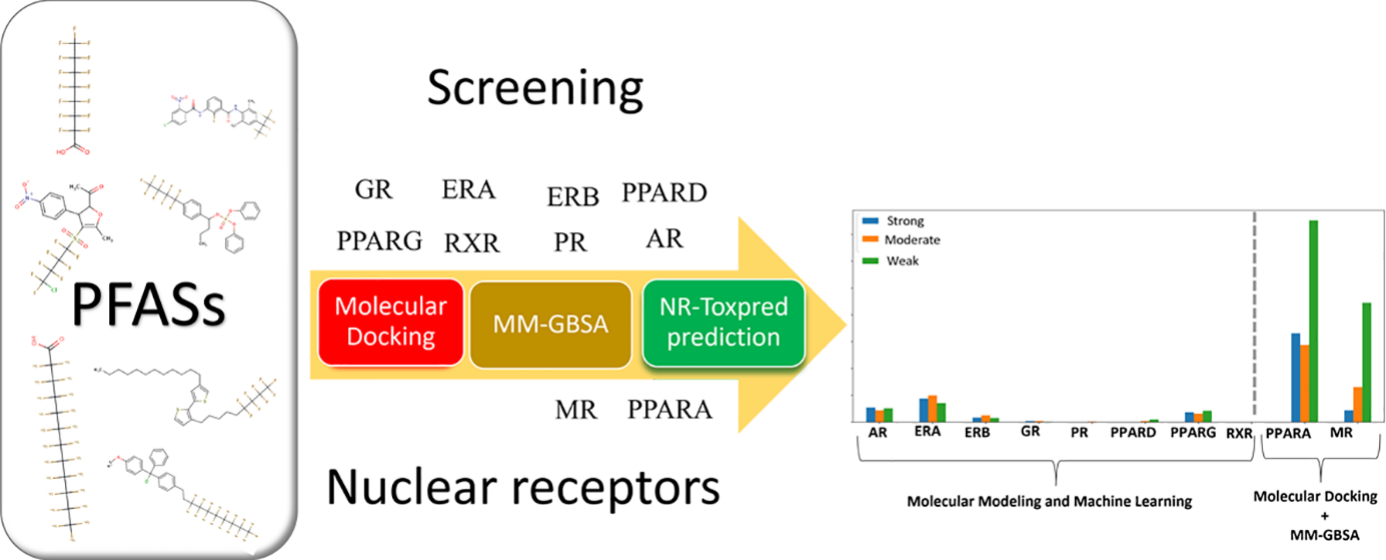

Per- and poly-fluoroalkyl substances (PFASs) are persistent, toxic chemicals that pose significant hazards to human health and the environment. Screening large numbers of chemicals for their ability to act as endocrine disruptors by modulating the activity of nuclear receptors (NRs) is challenging because of the time and cost of in vitro and in vivo experiments. For this reason, we need computational approaches to screen these chemicals and quickly prioritize them for further testing. Here, we utilized molecular modeling and machine-learning predictions to identify potential interactions between 4545 PFASs with ten different NRs. The results show that some PFASs can bind strongly to several receptors. Further, PFASs that bind to different receptors can have very different structures spread throughout the chemical space. Biological validation of these in silico findings should be a high priority.

## Abstract 2

Machine learning and other computational screening of PFASs identifies potential endocrine disruptors, informing risk assessment for human health and environmental policy on these persistent toxic chemicals.

## Introduction

Per- and poly-fluoroalkyl substances (PFASs) are a group of synthetic chemicals used in consumer products such as clothing and furniture for their water- and stain-repellent properties.^1,2^ Studies have identified PFASs in various environmental settings including groundwater,^3^ dust,^4^ and edible fish.^5^ Their persistence in the environment leads to exposure and accumulation in the human body over time.^3,6−9^ Researchers have detected perfluorooctanesulfonic acid (PFOS), perfluorooctanoic acid (PFOA), perfluorohexanesulfonic acid, and perfluorononanoic acid in human blood samples^10,11^ and breast milk.^12^ Many thousands of PFASs exist, but only a smaller subset has widespread commercial use.^13^ Despite continuous innovation in this field and the widespread environmental and biological presence of these substances and their metabolic and chemical degradation moieties, a significant gap persists in our understanding of their effects on human health.

The interactions of PFASs with biological molecules such as proteins and membranes have been the subject of extensive research.^14−19^ A recent review elucidated these interactions, shedding light on the molecular mechanisms through which PFASs can affect cellular processes.^14^ Previous studies have also highlighted the potential risks PFASs pose to human health, linking them to developmental toxicity,^20,21^ immunotoxicity,^22,23^ hepatotoxicity,^24^ and tumorigenesis.^25^ Studies also associate PFASs with lower bone mineral density^26^ and identify them as potential endocrine-disrupting chemicals.^19,21,27−29^ Interference with the activity of native hormones may therefore play an important role in the adverse effects of PFASs.

Guo et al., utilized surface plasmon resonance biosensing to assess the estrogenic activity of PFASs based on the ligand-induced conformation state of human estrogen receptor–alpha (ERA).^15^ Among the tested PFASs, only the 8-carbon compounds PFOS and PFOA showed binding to ERA, with weak estrogenic activity (PFOS stronger than PFOA). In contrast, the shorter perfluorobutanoic acid, perfluorobutanesulfonate, and longer perfluorododecanoic acid (PFDoA), perfluorotetradecanoic acid (PFTeDA) did not display significant binding or estrogenic activity.^15^ Rats and mice exposed to PFOA and PFOS show activation of Peroxisome proliferator-activated receptor alpha (PPARA).^16,30^ Rosen et al. proposed that PFOA activates a constitutive androstane receptor in PPARA knockout mice.^31^ Bjork et al. demonstrate that PFOS and PFOA exposure in rodents triggers multiple nuclear receptor (NR) activities and significantly alters hepatic triglyceride accumulation in liver cells.^32^

The two most widely used and studied PFASs, PFOS and PFOA, have been associated with adverse effects on reproductive^33^ and endocrine systems.^21^ While many studies have identified the activity of PFASs at high concentrations with different NRs,^15,16,30,34^ it is essential to prioritize those PFASs that demonstrate strong binding ability to different NRs even at lower concentrations. There are varying results regarding PFASs and their potential to disrupt endocrine functions, some studies indicate that certain PFAS compounds might impact NR activity,^15,16,30,34^ while others do not.^35^

Although PFOA and PFOS production have been restricted in many countries, hundreds, if not thousands of other PFASs are still utilized in consumer products and remain unstudied.^36,37^ Due to the prohibitive cost of testing all these chemicals, computational screening and shortlisting emerge as pragmatic approaches to prioritize the chemicals for in vitro and in vivo testing. Recently, Cheng and Ng utilized machine learning to classify the bioactivity of numerous PFASs, emphasizing the indispensable role of computational methods in understanding and predicting the impacts of these diverse compounds.^38^ Ng and Hungerbuehler explored the bioconcentration of PFASs, highlighting the complex interactions with proteins such as serum albumin and fatty acid binding proteins.^39^ Another study employed molecular docking to predict the bioaccumulation potential of PFASs.^40^ Molecular dynamics simulations were also used to predict the protein affinity of novel PFASs, providing insights into the bioaccumulation potential of emerging PFASs.^41^ Kwon et al. developed innovative semisupervised machine-learning models to predict the bioactivities of PFASs, offering an efficient and cost-effective alternative to traditional bioactivity assessments and providing insights into the bioactive properties of these widely used substances.^42^ In our previous study, we utilized machine learning and molecular modeling to prioritize PFAS chemicals for activity against androgen receptor (AR) and experimentally verified some of the PFASs as competitive antagonists of AR.^27,43^

In this study, we take advantage of in silico molecular screening and machine learning to identify PFASs that may strongly interact with several key NRs, including AR, ERA, estrogen receptor–beta (ERB), glucocorticoid receptor (GR), progesterone receptor (PR), PPARA, peroxisome proliferator-activated receptor delta (PPARD), peroxisome proliferator-activated receptor gamma (PPARG), mineralocorticoid receptor (MR), and retinoid X receptor alpha (RXRA). Our findings aim to illuminate the potential risks of unstudied PFASs and guide subsequent research and regulatory action.

## Materials and Methods

### PFAS Data Set Curation and Preparation for Molecular Modeling Studies

This study explored the potential use of machine learning and high-throughput virtual screening to swiftly screen PFASs for their possible binding to ten NRs. SMILES data for PFASs were downloaded on 15th October 2019 at 12:58 PM from the EPA (Environmental Protection Agency, USA) CompTox Chemicals Dashboard (https://comptox.epa.gov/dashboard), which had a total of 6330 PFASs. The downloaded date is noted due to the regular updates made to the Dashboard. Those PFASs without SMILES codes were excluded from the downloaded data set. In this study, we adopted specific criteria for the inclusion of PFASs, influenced by the evolving understanding and categorizations of these compounds as evidenced by international bodies and research literature.^44,45^ SMILES without perfluorinated units or having fewer than three total fluorine atoms were removed. After this, duplicates were removed based on their Inchi-key. The remaining 4545 PFASs were prepared for analysis using the Schrodinger suite^46^ LigPrep module by generating ionization, tautomeric states, and stereoisomers at pH 7.4, with a maximum of 32 states for each PFAS. Each PFAS tautomer, ionization variant, and stereoisomer state was treated as a unique structure, which was then energy minimized using the optimized potentials for liquid simulations (OPLS3e) force field with default parameters.^47^

### Molecular Docking of Reference Ligands and PFASs to NRs

This study employed an ensemble docking procedure to identify the binding poses of PFASs in the ligand binding pocket of the ligand binding domain of various NRs (AR, ERA, ERB, GR, PR, PPARA, PPARG, PPARD, MR, and RXRA). We obtained several structures for each of the ten NRs from the Protein Data Bank, with specific details about these structures outlined in Table S1, Supporting Information. The criteria for selecting these structures were based on factors such as the resolution of the structure (with a preference for higher-resolution structures) and the diversity in the range of ligand structures (to account for various ligand-binding modes). The crystal structures were then prepared by removing crystallographic water molecules, adding all hydrogen atoms to the protein, allocating bond orders, and minimizing energies using the protein preparation wizard in the Schrodinger software suite. We generated additional structures from the prepped crystal structures using MD simulation and induced fit docking procedures as described in our previous work on AR,^43^ to collectively make an ensemble suite of structures for each NR. Using the Glide module in the Schrodinger suite, we generated a grid box of size 10 Å × 10 Å × 10 Å for each of the NRs and centered this box on the center of mass of the cocrystallized ligand. A set of endogenous and related ligands known to bind to NRs (hereafter referred to as reference ligands) and our curated PFASs ligand set were then docked to the ensemble conformations of ten NRs using the Glide XP algorithm^48−50^ and default Glide settings. The single point MM-GBSA^51,52^ free energy of binding was calculated using the AMBER 18^53^ software suite for each of the docked NR–PFASs complexes (see details in the Supporting Information).

### Machine-Learning Predictions using NR-ToxPred

Recently, we developed NR-ToxPred (http://nr-toxpred.cchem.berkeley.edu/),^54^ which features a series of machine-learning models for predicting chemicals binding to eight NRs (AR, ERA, ERB, GR, PR, PPARG, PPARD, and RXRA). In this context, we employed NR-Toxpred to predict the activity of PFASs utilizing binding class models for AR, ERA, ERB, GR, PR, and PPARG using Morgan fingerprints and the SuperLearner algorithm. For PPARD and RXRA, we used an effector model, again employing Morgan fingerprints and the SuperLearner algorithm. The applicability domain options were configured with default settings: the minimum number of chemicals, denoted *N*_min_, was set to 1, and the similarity cutoff *S*_cutoff_, was established at 0.25. The applicability domain provides the reliability of the model for the predictions.

### Shortlisting and Classification of PFASs

For each NR, docking results were combined for each PFAS chemical. PFASs were shortlisted if their docking scores and Δ*G*_bind_ values were within the 10% threshold of the maximum values found for the reference ligand data for the respective receptor. The shortlisted chemicals were filtered through NR-Toxpred machine-learning models to predict if the chemical is an active agonist or antagonist for AR, ERA, ERB, GR, PR, and PPARG. For PPARD and RXRA, we used the NR-Toxpred effector model to predict if the chemical is active or inactive. For MR and PPARA, NR-Toxpred predictions were not available, so we used molecular docking and MM-GBSA (Δ*G*_bind_) free energy to shortlist their potential binders.

We calculated % change in the docking score and Δ*G*_bind_ as follows
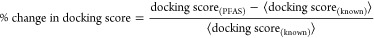
1
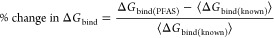
2
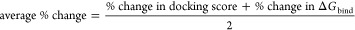
3

We classify the binding strength based on the average % change as follows:

- “Strong binder”: if the average % change is >0%,
- “Moderate binder”: if the average % change is between −10 and 0% and
- “Weak binder”: if the average % change is ≤ −10%.

## Results and Discussion

### Performance of Docking on Reference Ligands

We began by validating the molecular docking protocol, which involved docking the reference ligands for each receptor to the ligand-binding domain of 10 NRs (Table S2, Supporting Information). We compared the binding poses of the reference ligands from the docking protocol to the cocrystallized structures of each receptor. Figure S1 displays the superimposed binding poses from docking against the cocrystallized structures for reference ligands. The docking poses closely matched the X-ray crystal structures, with RMSDs of approximately 0.8 Å for each NR. Thus, these results confirm the efficacy of our novel ensemble molecular docking protocol for screening chemicals against NRs. Building on this validated protocol, we proceeded to predict the binding of PFASs to various NRs.

### Comparison of Computational Predictions with Experimental Data for Perfluoroalkyl Substances across Different Receptors

We compared our predictions for various receptors (ERA, ERB, PPARD, and PPARA) with the available experimental data from the literature.^34,55−57^ The details of this comparison can be found in the Supporting Information Tables S3–S6. Our computational predictions generally reveal a trend of moderate to weak binding affinities, with docking scores ranging mainly from −11 to −6 (kcal/mol). Furthermore, Δ*G*_bind_ shows a very weak binding for all the receptors. Interestingly, while our predictions align with experimental data in terms of relative binding affinities, discrepancies arise when we incorporate the NR-toxpred (machine learning) results and cutoffs for the docking score and Δ*G*_bind_ energy. Such differences can be attributed to the inherent variations between in silico methodologies and experimental conditions.

For the ERA and ERB receptors, we calculated *R*^2^ values of 0.56 and 0.61, respectively, for IC_50_ vs docking score, suggesting a moderate correlation. We observed a stronger correlation for both ERA and ERB, with *R*^2^ values of 0.81 and 0.72 for IC50 vs MMGBSA free energy, respectively. This emphasizes the importance of considering free energy predictions alongside docking scores, offering a more comprehensive perspective on the potential binding interactions. Many PFASs are predicted to be inactive using the combined approach, which corresponds with the high concentrations needed for activity, as seen in the experimental data.^34,55−57^ While experimental data may show activity, such findings are often noted at considerably elevated concentrations, which might surpass actual exposure levels at in vivo scenarios.^58,59^ This accentuates the pivotal role of computational tools in establishing preliminary screening thresholds and emphasizes the importance of considering typical exposure scenarios in interpreting results. Given these findings, we comprehensively screened PFASs against various NRs.

### Screening of the PFASs against NRs

Details of the number of PFAS chemicals binding to the NRs are provided in Table 1. Docking scores, MM-GBSA (Δ*G*_bind_), and predicted chemical binding activity for all receptors are available as Microsoft Excel files in the Supporting Information. Our NR-Toxpred results indicate that ERA is the NR with the highest number, 257, of predicted actives. Out of these, 43 chemicals were predicted to be both ERA agonists and antagonists (Ago-Ant) by our NR-Toxpred machine-learning model. Additionally, 159 PFASs were predicted as ERA antagonists and 55 as agonists (Table 1). The next highest number of actives was found for AR, with 149 PFASs predicted as active, with 104 of these predicted to be antagonists and 33 predicted as agonists (Table 1) by our NR-Toxpred machine-learning models. The third highest number of actives was for PPARG, with 111 PFASs predicted to be active (Table 1). In contrast, none of the PFASs were predicted to be active against RXRA, consistent with the recent study by Houck et al.,^60^ except for the perfluoro-2,5-dimethyl-3,6-dioxanonanoic acid (DTXSID00892442) and 7:3 Fluorotelomer alcohol (DTXSID50382621), which showed very weak in vitro interactions with RXRA.^60^ The docking and MM-GBSA scores for DTXSID50382621 and DTXSID00892442 are −8.55 and −36.42, and −7.53 and −41.78 kcal/mol, respectively, suggesting that they might be experimental outliers or extremely weak binders. RXRA was the focus over other RXR isotypes because it is very widely expressed and has the best structural and training data. The number of actives for ERB, GR, and PR was between 58 and 9, indicating that less than 1% of the PFASs screened were predicted to be active against these receptors. For PPARD, only 13 chemicals were predicted to be active by using molecular modeling and machine-learning techniques. We do not have an NR-ToxPred machine-learning model for PPARA and MR, so we had to rely only on molecular docking and Δ*G*_bind_ values to identify the active PFASs, which is considerably less restrictive. Employing these approaches, 1371 and 620 PFASs were predicted to be binders of PPARA and MR, respectively, possessing better docking and Δ*G*_bind_ scores than the average scores of the reference ligands (eq 3). Given that PPARA is an established target of many PFASs, it is perhaps not surprising that this number of predicted binders is large; however, there may yet be some false positives due to the lack of filtering by machine learning.

**Table 1:** Total Number of Predicted Active PFAS Chemicals for 10 Nuclear Receptors

receptor	antagonist–agonist	antagonist	agonist	total actives
Molecular Modeling and Machine Learning				
AR	12	104	33	149
ERA	43	159	55	257
ERB	6	51	1	58
GR		9		9
PR		2		2
PPARG	4	104	3	111
PPARD				13
RXR				0
Molecular Modeling (Molecular Docking and MM-GBSA)				
PPARA				1371
MR				620

Next, we classified the binding as strong, moderate, or weak based on the average percent change in docking score and Δ*G*_bind_ of each PFASs relative to the average docking score and Δ*G*_bind_ of reference ligands of each receptor. The distribution of the number of PFASs by binding affinity to each receptor is presented in Supporting Information Figure S2. Specifically, 54, 87, 17, 4, 37, 44, and 331 PFASs showed stronger binding values compared to the reference ligands for AR, ERA, ERB, GR, PPARG, MR, and PPARA, respectively (Figure S3, Supporting Information). 44, 99, 25, 4, 2, 4, 32, 288, and 131 PFASs exhibited moderate binding to AR, ERA, ERB, GR, PR, PPARD, PPARG, PPARA, and MR, respectively. AR, ERA, ERB, GR, PPARD, PPARG, PPARA, and MR have 51, 71, 16, 1, 9, 42, 752, and 445 PFASs as weak binders. These data indicate that PPARA, PPARG, and ERA are the three most probable NR targets for PFASs of the ten receptors studied.

2D interaction diagrams and lists of the top five PFASs and reference ligands with AR, ERA, ERB, GR, PPARG, PPARD, PR, PPARA, and MR are depicted in Figures S4–S12 and Table 2, respectively. We also prepared interaction fingerprints between the receptors, the respective reference ligands, and the shortlisted PFAS chemicals. These fingerprints represent the presence or absence of a specific interaction between the ligand and a particular residue in the receptor’s binding site. The interaction fingerprint maps for reference ligands and the shortlisted PFAS chemicals against AR are shown in Figure S4, Supporting Information. This figure shows that PFAS to amino acid interactions at the binding site were similar to those of the reference ligands. For instance, the PFAS 4′-dodecyl-3-(6,6,7,7,8,8,9,9,9-nonafluorononyl)-2,2′-bithiophene (DTXSID20844584) exhibits interaction with hydrophobic residues including Gln711, Gly708, and Leu704 in the same fashion as the reference ligand (see Figure S4, Supporting Information).

**Table 2:**
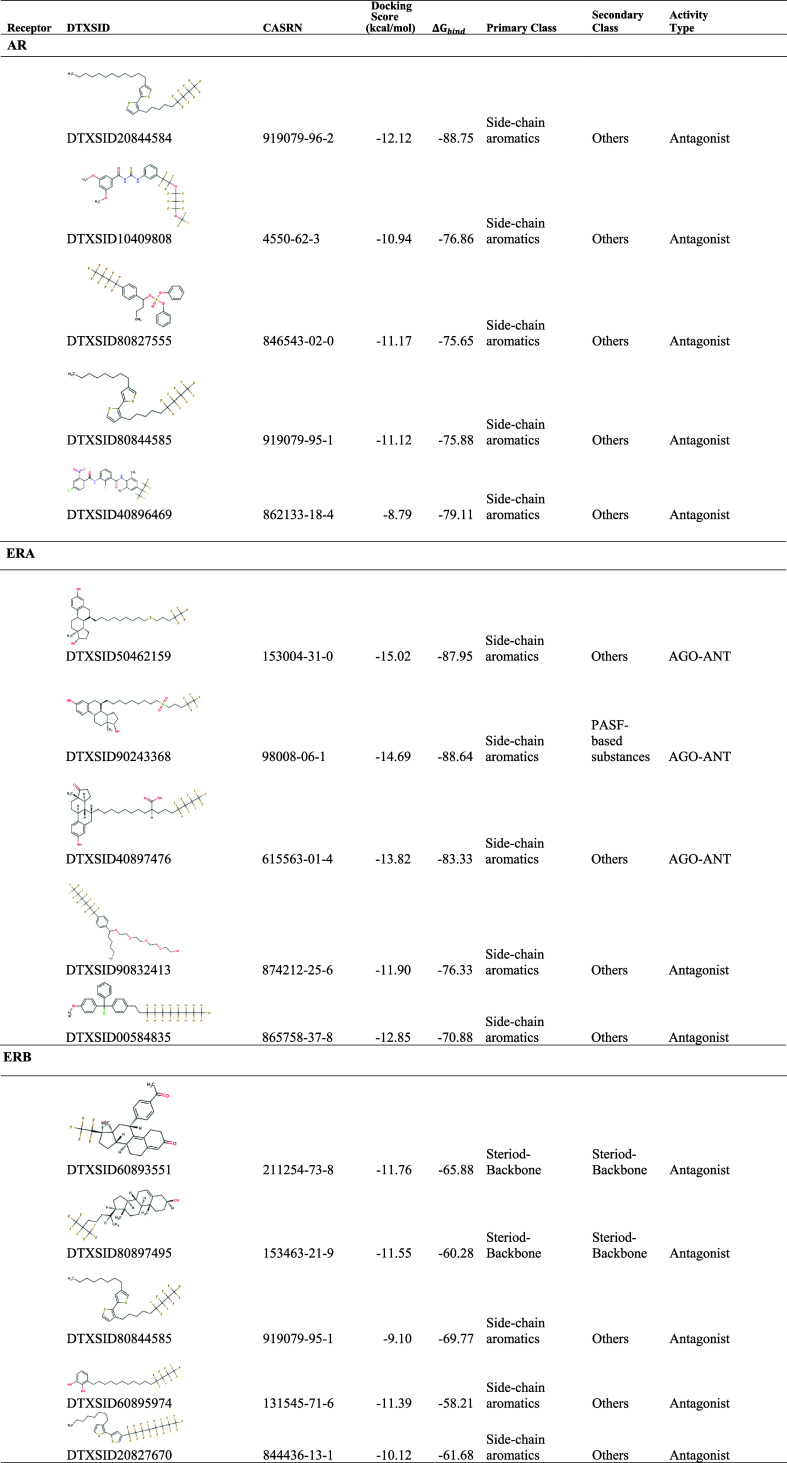

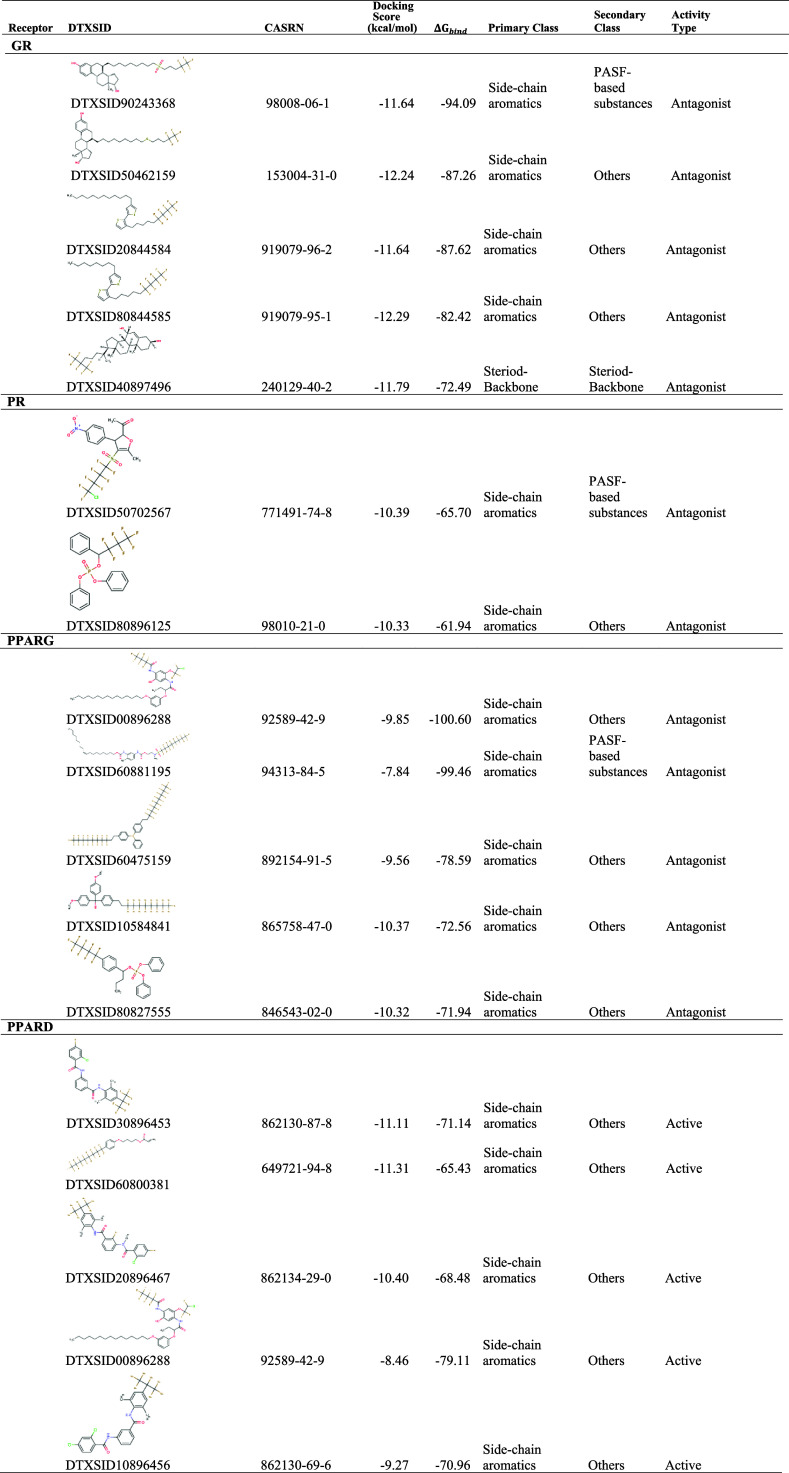

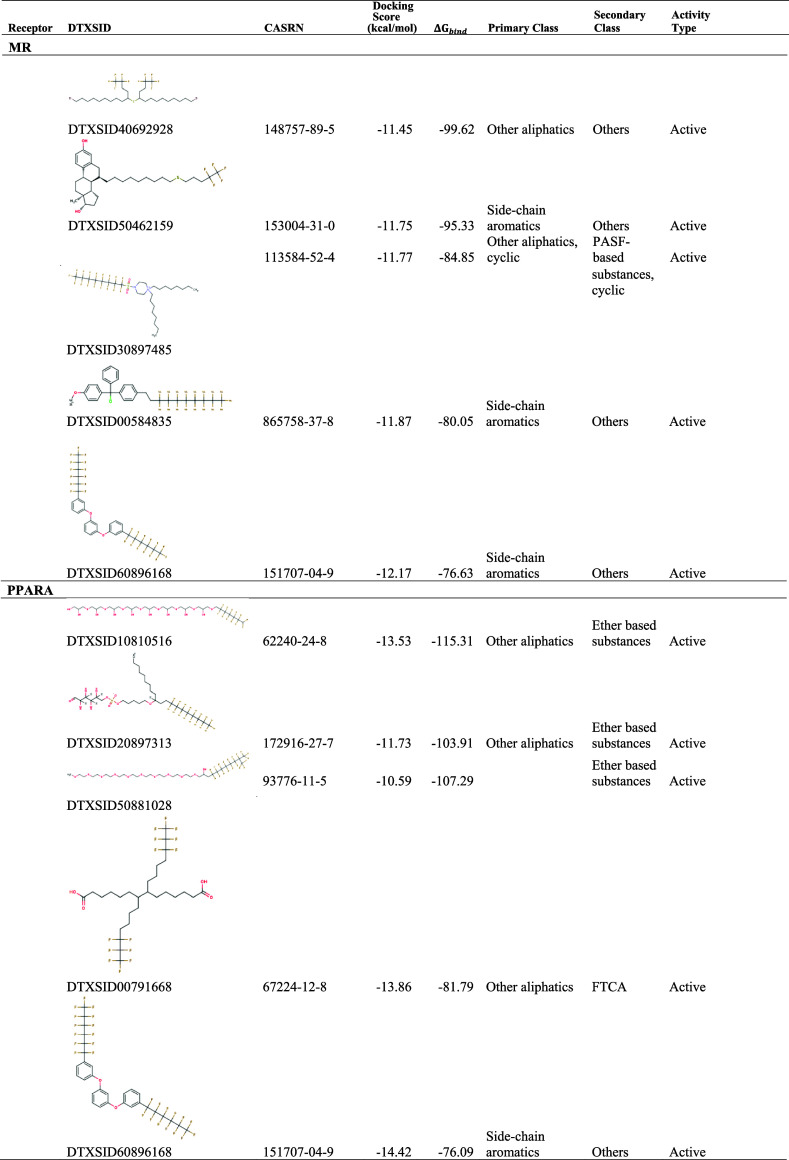
Top 5 Shortlisted PFASs for Different NRs

The top 5 PFASs predicted to bind to ERA are (1) *S*-deoxo fulvestrant (DTXSID50462159), (2) fulvestrant sulfone (DTXSID90243368), (3) (αR,7α)-3-hydroxy-α-((perfluorobutyl)propyl)-17-oxo-estra-1,3,5(10)-triene-7-decanoic acid (DTXSID40897476), (4) 13-[4-(Tridecafluorohexyl)phenyl]-3,6,9,12-tetraoxaoctadecan-1-ol (DTXSID90832413), and (5) 1-(chloro[4-(2-(perfluorooctyl)ethyl)phenyl]phenylmethyl)-4-methoxybenzene (DTXSID00584835). DTXSID90243368, DTXSID50462159, and DTXSID40897476 each have a steroid-like moiety, including a functional group that forms a hydrogen bond with Glu353 (Figure S5, Supporting Information). These results are consistent with previous studies on ERA and hormone binding.^61,62^*S*-Deoxo fulvestrant is an impurity of fulvestrant, the parent chemical of fulvestrant sulfone. Notably, fulvestrant sulfone is a well-known selective ER downregulator that has been extensively studied.^61,63−65^

The top 5 PFASs predicted to bind to the ERB are (1) Lonaprisan (DTXSID60893551), (2) (3β)-25,26,26,26,27,27,27-heptafluoro-cholest-5-en-3-ol (DTXSID80897495), (3) 3-(6,6,7,7,8,8,9,9,9-nonafluorononyl)-4′-octyl-2,2′-bithiophene (DTXSID80844585), (4) 3-(12,12,13,13,14,14,15,15,15-nonafluoropentadecyl)benzene-1,2-diol (DTXSID60895974), and (5) 4′-(heptadecafluorooctyl)-3-octyl-2,2′-bithiophene (DTXSID20827670). These PFASs are predicted to be strong binders with antagonist activity. Their docking scores and Δ*G*_bind_ values fall between −11.76 and −9.10 and between −65 and −58 kcal/mol, respectively. These top ERB-binding PFASs have either a steroid-like moiety (DTXSID60893551 and DTXSID80897495) or have side-chain aromatics (DTXSID80844585, DTXSID60895974, and DTXSID20827670), and all have extensive hydrophobic contacts with the receptor. For example, DTXSID60893551 shows hydrophobic interactions with multiple residues, including Ala302, Gly472, and Leu476. Similar hydrophobic interactions were observed for DTXSID80897495, DTXSID80844585, DTXSID60895974, and DTXSID20827670. DTXSID80897495 and DTXSID60895974 also each form a hydrogen bond with Glu305 (Figure S6, Supporting Information). DTXSID80844585 and DTXSID20827670 lacked this hydrogen bond interaction with GLU305, suggesting potential differences in their binding modes within the binding pocket. In addition, we note that previous studies show that DTXSID60895974 can trigger an allergic response in pentadecylcatechol (PDC)-sensitized mice.^66^

These PFASs are predicted to be antagonists for GR: fulvestrant sulfone (DTXSID90243368), *S*-deoxo fulvestrant (DTXSID50462159), 4′-dodecyl-3-(6,6,7,7,8,8,9,9,9-nonafluorononyl)-2,2′-bithiophene (DTXSID20844584), 3-(6,6,7,7,8,8,9,9,9-nonafluorononyl)-4′-octyl-2,2′-bithiophene (DTXSID80844585), and 25,26,26,26,27,27,27-heptafluorocholest-5-ene-3β,7α-diol (DTXSID40897496). DTXSID90243368 binds in a similar fashion to the reference ligands, forming hydrophobic interactions with various amino acid residues such as Ala605, Asn564, Cys736, Gln570, Gln642, Leu563, Leu608, Leu732, Met560, Met601, Met604, Met646, Phe623, and Tyr735. The shortlisted PFASs DTXSID50462159 and DTXSID40897496 form a hydrogen bond with Asn564 (see Figure S7, Supporting Information).

DTXSID90243368 has a steroid-like moiety, and the other four shortlisted PFASs for GR have side-chain aromatics. Consequently, they can all bind in a manner akin to that of the reference ligands by establishing hydrophobic interactions with GR residues, including Ala605, Asn564, Cys736, Gln570, Gln642, Leu563, Leu608, Leu732, Met560, Met601, Met604, Met646, Phe623, and Tyr735. These shortlisted PFASs also each form hydrogen bonds with Asn564 and Gln642 (see Figure S7, Supporting Information).

The top five PFASs predicted to bind to the PPARG receptor are (1) *N*-[5-(2-chloro-1,1,2-trifluoroethoxy)-2-hydroxy-4-{2-[3-(pentadecyloxy)phenoxy]butanamido}phenyl]-2,2,3,3,4,4,4-heptafluorobutanamide (DTXSID00896288), (2) 2-[(1,1,2,2,3,3,4,4,5,5,6,6,7,7,8,8,8-heptadecafluorooctane-1-sulfonyl)(methyl)amino]ethyl (9*Z*)-octadec-9-en-1-yl (4-methyl-1,3-phenylene)biscarbamate (DTXSID60881195), (3) bis[4-(3,3,4,4,5,5,6,6,7,7,8,8,9,9,10,10,10-heptadecafluorodecyl)phenyl](phenyl)phosphane (DTXSID60475159), (4) [4-(3,3,4,4,5,5,6,6,7,7,8,8,9,9,10,10,10-heptadecafluorodecyl)phenyl]bis(4-methoxyphenyl)methanol (DTXSID10584841), and (5) 1-[4-(nonafluorobutyl)phenyl]butyl diphenyl phosphate (DTXSID80827555). Each of these contains the side-chain aromatic ring favoring hydrophobic interactions. 2D interaction diagrams and interaction fingerprints show that all the shortlisted PFASs have receptor interactions similar to those seen with reference ligands (see Figure S8, Supporting Information).

The top 5 shortlisted PFASs against PPARD have a wide range in the average percentage change of the docking score and Δ*G*_bind_ relative to the reference ligands. For instance, 4-[4-(heptadecafluorooctyl)phenoxy]butyl prop-2-enoate (DTXSID60800381) had a relatively high docking score of −11.308 and MM-GBSA of −65.433, indicating a strong interaction with PPARD. In contrast, *N*-[5-(2-chloro-1,1,2-trifluoroethoxy)-2-hydroxy-4-{2-[3-(pentadecyloxy)phenoxy]butanamido}phenyl]-2,2,3,3,4,4,4-heptafluorobutanamide (DTXSID00896288) had a lower docking score of −8.461 and MM-GBSA of −79.11, suggesting a weaker interaction. However, NR-Toxpred data suggest that these are both active and are thus included in our predicted list. Also, all of these shortlisted PFASs interact with PPARD similarly to that of the reference ligands, including favorable interactions with PPARD hydrophobic residues: Val341, Cys285, ILE326, Leu330, and Met453 (see Figure S9, Supporting Information).

For PR, only two PFASs are predicted to be active antagonists, 1-[4-(4-chloro-1,1,2,2,3,3,4,4-octafluorobutane-1-sulfonyl)-5-methyl-3-(4-nitrophenyl)-2,3-dihydrofuran-2-yl]ethan-1-one (DTXSID50702567) and 2,2,3,3,4,4,4-heptafluoro-1-phenylbutyl diphenyl phosphate (DTXSID80896125). The 2D interaction diagrams and interaction profiles of DTXSID50702567 and DTXSID80896125 with PR can be found in Figure S10, Supporting Information. The 2D interaction diagrams reveal that both have similar interactions as those of the reference ligands including hydrophobic interactions with PR residues Cys891, Leu715, Leu718, Leu721, Leu763, Leu797, Leu887, Met756, Met759, Met909, Phe778, Phe794, Thr894, Trp755, and Tyr890.

Interactions of PFASs with the peroxisome PPARA are of considerable interest. Our top 5 predicted PFASs are 34,34,35,35,36,36,37,37,38,38,39,39-dodecafluoro-4,8,12,16,20,24,28,32-octaoxanonatriacontane-1,2,6,10,14,18,22,26,30-nonol (DTXSID10810516), and galactose-6-[5-[(3(-perfluorooctyl)-1-nonylpropyl)oxy]pentylhydrogen phosphate] (DTXSID20897313). 32-(Perfluoro-7-methyloctyl)-2,5,8,11,14,17,20,23,26,29-decaoxadotriacontane-31-ol (DTXSID50881028), 7,8-bis(5,5,6,6,7,7,7-heptafluoroheptyl)tetradecanedioic acid (DTXSID00791668) and 1,1′-[1,3-phenylenebis(oxy)]bis[3-(tridecafluorohexyl)benzene] (DTXSID60896168). These also show similar binding interactions with PPARA as those seen for the reference ligands (see Figure S11, Supporting Information).

The 2D interaction diagrams for the top five PFASs predicted to be active against MR are given in Figure S12, Supporting Information. Analysis of the ligand interaction illustrates that DTXSID40692928, DTXSID50462159, DTXSID30897485, DTXSID00584835, and DTXSID60896168 engage in hydrophobic interactions with MR residues, including Ala773, Asn770, Cys942, Gln776, Leu766, Leu769, Leu772, Leu814, Leu938, Met807, Met845, Met852, Phe829, Phe941, Ser810, Thr945, and Trp806. For the top 5 chemicals, the docking score and Δ*G*_bind_ (kcal/mol) span from −12.19 and −76.63 for DTXSID60896168 to −11.45 and −99.62 for DTXSID40692928.

### Commercially Relevant PFASs

The list of commercially relevant PFASs^13^ shortlisted for predicted AR, ERA, PPARG, PPARA, and MR binding is tabulated in Table S7 (see the Supporting Information), and 2D interaction diagrams for the top 5 commercially relevant PFASs are provided in Figures S13–S17, Supporting Information. For the AR, nine commercially important PFASs were predicted to be active. Notably, *N*-ethyl-*N*-[2-(phosphonooxy)ethyl]perfluorooctanesulfonamide was predicted to be a strong, active antagonist with a docking score and Δ*G*_bind_ of −9.22 and −60.24 kcal/mol, respectively. Three PFASs were predicted to be moderately active: (1) 2-(perfluorodecyl)ethyl acrylate (agonist), (2) *N*-methylperfluorooctanesulfonamidoethyl acrylate (agonist–antagonist), and (3) 2-((ethyl(pentadecafluoroheptyl)sulfonyl)amino)ethyl acrylate (antagonist). Others were predicted to be weakly active.

Twelve commercially important PFASs were predicted to be active against the ERA, with docking scores ranging from −9 to −7.5 and Δ*G*_bind_ energy ranging from −50 to −42 kcal/mol. Several PFASs, including Perfluorohexadecanoic acid (DTXSID1070800) and 2-(*N*-ethyl-*N*-(perfluorooctylsulfonyl)amino)ethyl acrylate (DTXSID3059975), demonstrated moderate activity. Additionally, *N*-methylperfluorooctanesulfonamidoethyl acrylate (DTXSID80865199) and perfluorotridecanoic acid (DTXSID90868151) were predicted as weak agonists and antagonists, respectively.

PFTeDA has been widely detected in the environment and various biota, with increasing concentrations over time, raising concerns about its potential ecological and human health impacts, including adverse effects on the male reproductive system by affecting regeneration of Leydig cells, which play a crucial role in testosterone production and sperm development.^67,68^ The presence of fluorotelomer alcohols and perfluoroalkyl sulfonamido ethanols, including *N*-methylperfluorooctanesulfonamidoethyl acrylate (DTXSID80865199), in the environment has been highlighted in recent studies.^69,70^ Moreover, our data predict this chemical as a potential binder for both AR and ERA.

In the case of PPARG, only a single commercially relevant PFAS, perfluorooctadecanoic acid (PFODA) (CASRN: 16517-11-6), was predicted to be a weak antagonist with a docking score of −8.22 and an Δ*G*_bind_ of −44.59 kcal/mol. Our data also predicted PFODA to be a strong binder for PPARA, which is consistent with previous experiments wherein PFODA induced the expression of PPARA and PPARG, but to a lesser extent.^71^ For PPARA, we predicted 51 different commercially important PFASs to be active with a docking score range from −14 to −7.5 and Δ*G*_bind_ range from −71 to −41 kcal/mol. Gestational exposure to certain PFASs, particularly PFOS, perfluorodecanoic acid, and PFDoA, is associated with higher risks of congenital heart defects in newborns.^72^ For MR, we predicted 14 commercially relevant PFASs to be active, of which three were classified as moderate while the rest of the PFASs were classified as weak. The moderate actives are (1) 2-(*N*-ethylperfluorooctanesulfonamido)acetic acid (DTXSID5062760), (2) 1,1,2,2-tetrahydroperfluorohexadecyl acrylate (DTXSID6067836), and (3) perfluorooctadecyl iodide (DTXSID9067514).

### Exploring the Chemical Space of PFASs

**Figure 1:**
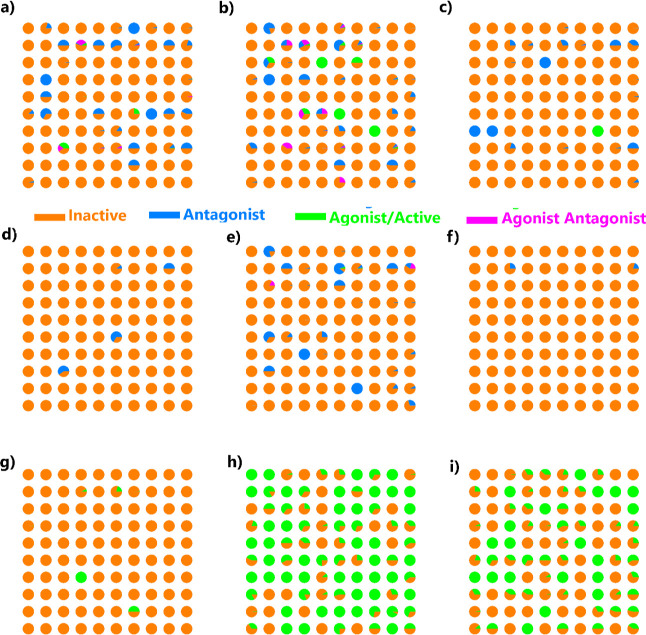
SOM projections of chemical space for PFASs. Each panel represents the distribution of various compounds with respect to their activity toward specific NRs predicted using machine-learning and molecular modeling techniques. (a) AR, (b) ERA, (c) ERB, (d) GR, (e) PPARG, (f) PR, (g) PPARD, and only molecular docking and single-point free energy calculations were employed to shortlist the PFASs for (h) PPARA and (i) MR receptors. The colors in each panel indicate the activity of the compounds: orange: inactive, blue: antagonist, green: agonist/active, pink: agonist/antagonist.

The chemical space of our PFASs data set was visualized using self-organizing maps (SOM) as depicted in Figure 1. SOMs were constructed utilizing Morgan fingerprints, also called extended-connectivity fingerprints (ECFP4), as descriptors using RDKIT (version 2021.09.3)^73^ and the Somoclu (version 1.7.5)^74^ libraries. From Figure 1, the majority of PFASs in our data set are categorized as nonbinders/inactive (represented in orange). Notably, those that do exhibit binding to the various NRs display diverse structures scattered throughout the chemical space.

**Figure 2:**
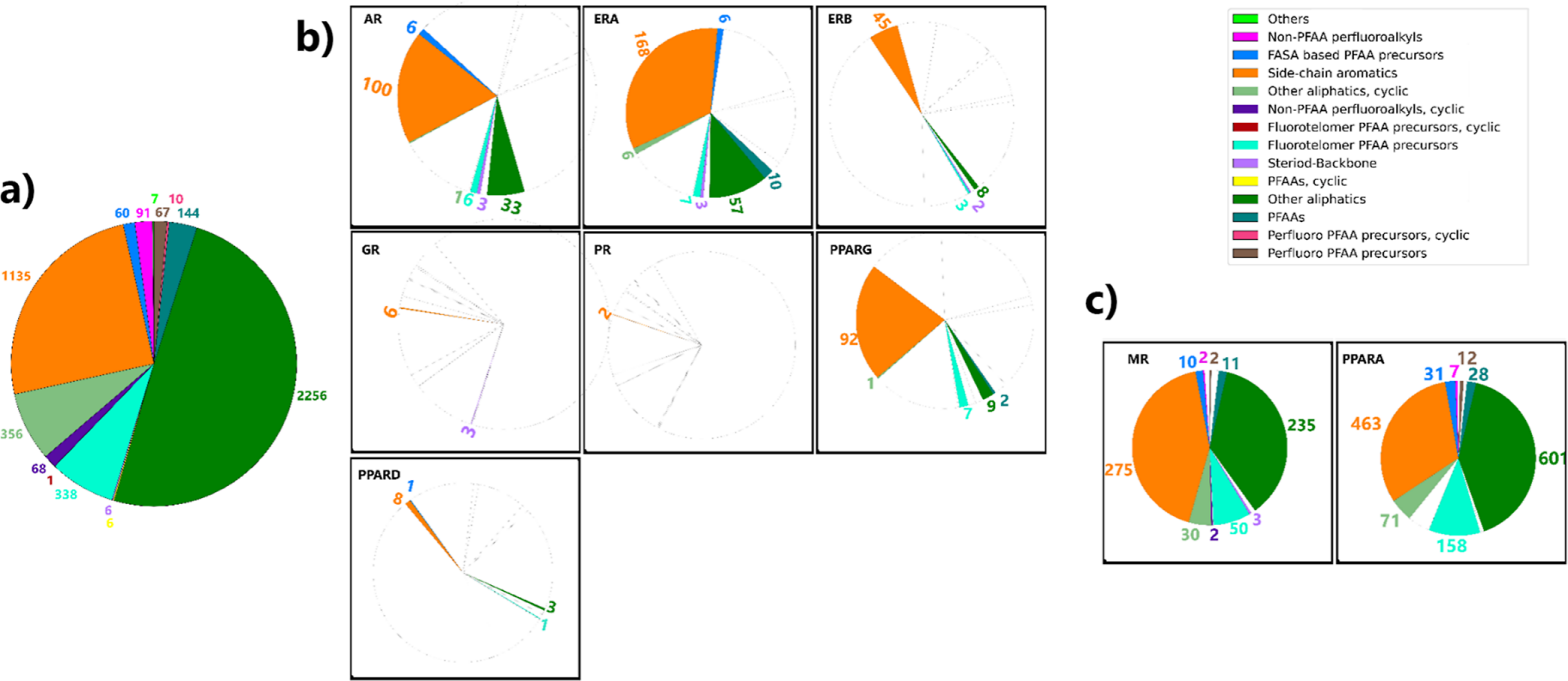
Primary chemical classification of PFASs (a) screened library: represents the initial collection of PFAS classified according to their chemical nature. The pie chart depicts the distribution of different chemical classes, with the respective counts highlighted on each segment. (b) Combined molecular modeling and machine learning - shortlisted PFASs’s classes for NRs: this section comprises multiple pie charts, each corresponding to a specific NR including AR, ERA, ERB, GR, PR, PPARG, and PPARD. Each chart displays the distribution of PFAS classes that were shortlisted for the respective receptor using a combination of molecular modeling and machine-learning approaches. (c) The pie charts depict the distribution of PFAS classes selected for MR and PPARA; due to the absence of machine-learning models, only molecular docking and single point free energy calculations were employed to shortlist the PFASs for MR and PPARA receptors. Abbreviations in legend Perfluoroalkyl acid (PFAA), perfluoroalkanesulfonamide (FASA).

The primary and secondary classes of PFASs^75^ for the screened data set and the shortlisted chemicals for each receptor are illustrated in Figure 2. Detailed tabulations for each receptor can be found in Tables S8–S16 of the Supporting Information. Figure 2a provides a primary class classification of the screened library of PFASs, which clearly shows that the “Other aliphatics” class is the dominant class in the entire library. From Figure 2b, it is evident that each NR prefers binding to PFASs characterized under the primary classes the “side-chain aromatics” and “Other aliphatics”. Notably, PFASs from the “Side-chain aromatics” class were significantly active across numerous receptors, especially AR, ERA, PPARG, and PPARA. “FASA-based PFAA precursors” and “Fluorotelomer PFAA precursors” exhibit interactions with multiple receptors, although their counts were relatively lower than those of side-chain aromatics. The AR predominantly interacts with chemicals classified under “FASA-based PFAA precursors”, especially the “*N*-Alkyl FASACs” secondary class. Additionally, notable interactions with the “Fluorotelomer PFAA precursors” and “Other aliphatics” classes were observed, particularly with compounds’ secondary class of “n:2 FTACs” and “PASF-based substances”, respectively. In contrast, the ERA demonstrated a pronounced affinity for the “Side-chain aromatics” class, especially those in the “Others” secondary class, while still has significant chemicals in “FASA-based PFAA precursors”, particularly the “*N*-Alkyl FASACs”. ERB showed a preference for the chemicals characterized as “Side-chain aromatics”, with the secondary class of “Fluorotelomer PFAA precursors” and “Other aliphatics”. GR and PR had a more limited chemical space, with the majority of shortlisted PFASs classified as “Side-chain aromatics”. Other aliphatic PFASs showed interactions with most NRs, with notably high counts for PPARA and MR (Figure 2c). A prominent secondary class of predicted binders of the NRs are PASF-based substances. There is therefore a strong argument for phasing out or banning side-chain aromatic PFASs.

In summary, PFASs, due to their potential interactions with some NRs, may act as endocrine and metabolic disruptors, thereby posing considerable risks to human health. In our study, we utilized molecular modeling and machine learning to investigate the interactions of a broad spectrum of PFASs with 10 distinct NRs. Our computational analysis suggests that certain PFASs may have the capacity to bind strongly to these varied NRs, exhibiting multiple binding modes. The PFASs identified as potential binders are widely spread throughout the chemical space and resist narrow classification. Furthermore, the NRs differ widely in terms of the range and specificity of the PFASs that bind to them. It is important to note that our findings are primarily predictive in nature. Therefore, biological validation of these in-silico predictions emerges as a critical next step. Such validation is essential not only for corroborating the potential binding effects but also for understanding the real-world implications of these interactions, especially in terms of their potential adverse biological impacts.

## Supporting Information Available

- Superimposed structures of the crystallographic ligand and its docked binding pose for receptors AR, ERA, ERB, GR, PPARD, PPARG, PR, RXR, MR, and PPARA; distribution of PFASs according to binding strength; 2D interaction diagrams of various receptor–ligand complexes; 2D interaction diagrams of top-scoring PFAS chemicals (identified by their DTXSID numbers) and different receptors; 2D interaction diagrams of top scoring commercially important PFAS different receptors; comprehensive information on NR structures, endogenous and known ligands, and their docking scores and binding energies; and primary and secondary class distribution of shortlisted PFASs against various receptors (PDF)
- Detailed information on all PFASs and NRs, such as docking scores, free energy of binding, and other relevant data (ZIP)
