# Supplementary material for: Prediction of the Interactions of a Large Number of Per- and Poly-Fluoroalkyl Substances with Ten Nuclear Receptors
Source: Environ Sci Technol. 2024 Feb 29;58(10):4487–99. doi: 10.1021/acs.est.3c05974 (PMC10938639; doi:10.1021/acs.est.3c05974)
Supplement: Supplementary file 1 — es3c05974_si_001.pdf [file es3c05974_si_001.pdf]

Prediction of the Interactions of a Large Number of Per- and Poly-Fluoroalkyl Substances (PFASs) with Ten Nuclear Receptors

Ettayapuram Ramaprasad Azhagiya Singam[1], Kathleen A. Durkin[1*], Michele A. La Merrill[2], J. David Furlow[3], Jen-Chywan Wang[4] and Martyn T. Smith[5*]

[1] Molecular Graphics and Computation Facility, College of Chemistry, University of California, Berkeley, CA 94720, USA

[2] Department of Environmental Toxicology, University of California, Davis, CA 95616, USA

[3] Department of Neurobiology, Physiology and Behavior, University of California, Davis 95616, CA, USA

[4] Department of Nutritional Sciences and Toxicology, University of California, Berkeley, CA 94720, USA

[5] Division of Environmental Health Sciences, School of Public Health, University of California Berkeley, CA 94720, USA

* To whom correspondence should be addressed. Tel: +1(510) 642-6719; Email:durkin@berkeley.edu

Keywords: PFASs, Nuclear Receptor,

Summary information:

Number of Pages: 51 pages

Text: S3

Tables: S4 – S34

Figures: S35 – S51

References: S52

Single Point MM-GBSA Free Energy Calculation

The single point MM-GBSA[1,2] free energy of binding was calculated using the AMBER 18[3] software suite for each of the docked NR - PFASs complexes. Partial atomic charges for each PFASs were calculated utilizing Antechamber employing the AM1-BCC method [4], whereas the AMBER FF14SB force field was used for the protein. Each complex was solvated using a TIP3P water box. These solvated complexes were energy minimized in four steps: 1) Minimization of the solute with a restraint weight of 500 kcal/mol/$Å^2$ for 1000 steps, 2) Minimization of the solute with a restraint weight of 100 kcal/mol/$Å^2$ for 1000 steps, 3) Minimization relaxing the solute with a restraint weight of 1 kcal/mol/$Å^2$ for 1000 steps, and 4) 2500 steps of steepest descent without any positional restraint. The MM-GBSA binding free energy (ΔG_{bind}) of each minimized complex structure was then calculated using an infinite cutoff (999 Å) and a dielectric protein constant of 4.

Table S1: PDB ID of NR structures selected for molecular docking with respective resolution and co-crystallized ligand.

Receptor	PDB ID	Resolution (Å)	Co-crystallized Ligand
AR	3ZQT	2.29	Testosterone
AR	5V8Q	1.44	4-[(2S,3S)-2-ethyl-3-hydroxy-5-oxopyrrolidin-1-yl]-2-(trifluoromethyl)benzonitrile
AR	1T7T	1.70	5-alpha-dihydrotestosterone
ERA	1XP1	1.80	(2s,3r)-2-(4-{2-[(3r,4r)-3,4-dimethylpyrrolidin-1-yl]ethoxy}phenyl)-3-(4-hydroxyphenyl)-2,3-dihydro-1,4-benzoxathiin-6- ol
ERA	6VIG	1.45	(9beta,11beta,17beta)-11-{4-[2-(dimethylamino)ethoxy]phenyl}estra-1,3,5(10)-triene-3,17-diol
ERA	6VPF	1.60	Clomifene
ERB	3OMQ	1.97	2-[(trifluoromethyl)sulfonyl]-1,2,3,4-tetrahydroisoquinolin-6-ol
ERB	4ZI1	2.10	2-(4-hydroxyphenyl)-7-methyl-3-phenyl-1H-inden-5-ol
GR	1M2Z	2.50	Dexamethasone
GR	3BQD	2.50	1-[(1R,2R,3as,3bs,10ar,10bs,11S,12as)-1,11-dihydroxy-2,5,10a,12a-tetramethyl-7-phenyl-1,2,3,3a,3b,7,10,10a,10b,11,12,12a-dodecahydrocyclopenta[5,6]naphtho[1,2-f]indazol-1-yl]-2-hydroxyethanone
GR	4CSJ	2.30	N-[(2S)-1-[[1-(4-fluorophenyl)indazol-4-yl]amino]propan-2-yl]-2,4,6-trimethyl-benzenesulfonamide
PR	1A28	1.80	Progesterone
PR	1SQN	1.45	(14beta,17alpha)-17-ethynyl-17-hydroxyestr-4-en-3-one
PR	3G8O	1.90	N~2~-[4-cyano-3-(trifluoromethyl)phenyl]-N,N-dimethyl-N~2~-(2,2,2-trifluoroethyl)-L-alaninamide
PPARA	7E5I	1.58	(2S)-2-[[3-[[3-fluoranyl-4-(4-fluoranylphenoxy)phenyl]methylcarbamoyl]-4-methoxy-phenyl]methyl]butanoic acid
PPARA	2P54	1.79	2-methyl-2-(4-{[({4-methyl-2-[4-(trifluoromethyl)phenyl]-1,3-thiazol-5-yl}carbonyl)amino]methyl}phenoxy)propanoic acid
PPARA	7BQ1	1.52	Palmitic acid
PPARG	2PRG	2.30	2,4-thiazolidiinedione, 5-[[4-[2-(methyl-2-pyridinylamino)ethoxy]phenyl]methyl]-(9cl)

PPARG	3AN3	2.30	(2S)-2-benzyl-3-(4-propoxy-3-{[({4-[(3S,5S,7S)-tricyclo[3.3.1.1~3,7~]dec-1-yl]phenyl}carbonyl)amino]methyl}phenyl)propanoic acid
PPARG	5F9B	2.25	Caulophyllogenin
PPARD	1Y0S	1.93	Gw2331
PPARD	2BAW	2.30	Vaccenic acid
PPARD	3GZ9	1.80	(2,3-dimethyl-4-{[2-(prop-2-yn-1-yloxy)-4-{[4-(trifluoromethyl)phenoxy]methyl}phenyl]sulfanyl}phenoxy)acetic acid
RXR	1FM6	2.10	(9cis)-retinoic acid
RXR	1FM9	2.10	(9cis)-retinoic acid
RXR	1G5Y	2.00	Retinoic acid
RXR	1K74	2.30	(9cis)-retinoic acid
RXR	1MV9	1.90	Docosa-4,7,10,13,16,19-hexaenoic acid
RXR	1MZN	1.90	4-[2-(5,5,8,8-tetramethyl-5,6,7,8-tetrahydro-naphthalen-2-yl)-[1,3]dioxolan-2-yl]-benzoic acid
RXR	2ACL	2.80	Retinoic acid
RXR	3FUG	2.00	(2E)-3-[4-hydroxy-3-(3,5,5,8,8-pentamethyl-5,6,7,8-tetrahydronaphthalen-2-yl)phenyl]prop-2-enoic acid
RXR	4K4J	2.00	(2E,4E,6Z,8E)-8-(3,4-dihydronaphthalen-1(2H)-ylidene)-3,7-dimethylocta-2,4,6-trienoic acid
RXR	6A5Z	2.95	(9cis)-retinoic acid
MR	2AA2	1.95	Aldosterone
MR	2OAX	2.29	Spironolactone

Table S2: Endogenous and known ligands used in this study.

Receptors	Names	Docking score	ΔG$_{bind}$(kcal/mol)
**AR**	17β-trenbolone	-12.562	-52.7597
	Bicalutamide	-11.623	-57.9968
	Dihydrotestosterone	-11.749	-57.6087
	Hydroxyflutamide	-9.817	-44.8942
	Methyltrienolone	-12.827	-57.0108
	Neburon	-7.906	-48.0901
	p-DDE	-9.844	-46.1807
	Testosterone	-11.728	-57.062
	Vinclozolin	-9.015	-43.5834
**ERA**			
	Sodium Estrone sulphate	-8.03579	-46.1179
	Estrone	-10.3056	-48.557
	Estetrol	-12.169	-49.6497
	Estriol	-11.6326	-47.6861
	17beta-Estradiol	-10.7982	-46.8095
**ERB**			
	Sodium Estrone sulphate	-7.95479	-53.6181
	17beta-Estradiol	-11.4335	-51.5498
	Estrone	-9.63817	-48.0095
	Estetrol	-12.595	-50.4551
	Estriol	-11.9607	-48.5086
**GR**			
	Dexamethasone	-15.2754	-65.2044
	Cortisol	-14.9752	-63.6798
	Prednisolone	-14.6199	-62.6903
	Corticosterone	-13.7057	-63.9094
	Prednisone	-13.6854	-61.9367
	Fosdagrocorat	-13.607	-89.4922
	GSK 9027	-13.5343	-78.6618

	AZD-5423	-12.2937	-74.5911
	RU-486	-11.8393	-74.3795
	Triamcinolone acetonide	-12.8025	-65.6924
	Deacylcortivazol	-15.5997	-84.254
**PR**			
	Drospirenone	-13.1842	-66.3313
	Dydrogesterone	-12.9912	-59.4461
	levonorgestrel	-11.4873	-60.1424
	norethisterone	-10.925	-61.5656
	Progesterone	-12.8514	-61.6711
**PPARA**			
	GW_7647	-10.883	-80.0372
	GW_6471	-10.604	-92.6266
	CP_775146	-10.261	-71.2839
	Oleylethanolamide	-9.028	-57.4425
	WY_14643	-8.686	-45.712
	Fenofibrate	-7.841	-50.901
	Palmitoylethanolamide	-7.84	-57.4585
**PPARD**			
	2-(4-{3-[1-[2-(2-chloro-6-fluoro-phenyl)-ethyl]-3-(2,3-dichloro-phenyl)-ureido]-propyl}-phenoxy)-2-methyl-propionic acid	-12.5498	-87.5648
	GW2331	-12.6467	-79.7926
	Vaccenic acid	-8.9151	-56.1392
	3,4,5-trisubstituted isoxazoles	-12.7562	-88.2153
	[(7-{[2-(3-morpholin-4-ylprop-1-yn-1-yl)-6-{[4-(trifluoromethyl)phenyl]ethynyl}pyridin-4-yl]thio}-2,3-dihydro-1h-inden- 4-yl)oxy]acetic acid	-10.3668	-96.6551
	GW0742	-12.3594	-62.7754

	GW501516	-12.1149	-60.3233
	Telmisartan	-7.99755	-69.248
**PPARG**			
	13-Hydroxyoctadecadienoic_acid	-8.67093	-54.9552
	15-deoxy-Î”12	-10.0701	-49.5362
	15-Hydroxyeicosatetraenoic_acid	-9.52845	-50.6346
	Farglitazar	-8.91537	-69.4443
	Pioglitazone	-10.5876	-55.7271
	Troglitazone	-10.8524	-68.0727
**MR**			
	21-Hydroxyprogesterone	-10.938	-59.1122
	Aldosterone	-13.821	-66.4896
	Apararenone	-10.165	-51.868
	Canrenone	-10.566	-63.5574
	Eplerenone	-11.202	-70.3637
	Finerenone	-9.329	-54.5058
	Fludrocortisone	-12.534	-65.0078
	Hydrocortisone	-12.344	-63.6743
	Spironolactone	-11.799	-70.7111
**RXR**			
	UVI3003 antagonist	-16.9707	-99.2418
	LG100268 agonist	-16.3161	-91.5232
	Bexarotene agonist	-16.442	-87.6966
	Docosahexaenoic acid agonist	-15.817	-94.9655
	Alitretinoin agonist	-14.427	-90.6552
	HX 531 antagonist	-14.3831	-80.0752

Table S3: Comparison of ERA Predictions with Experimental Data for Perfluoroalkyl Substances (PFASs)

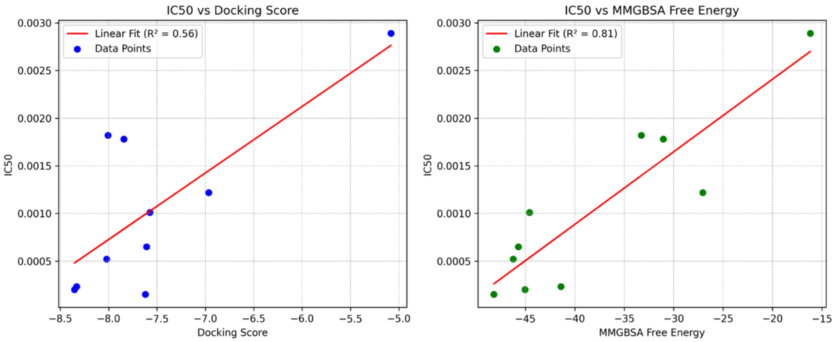

DTXSID	NAMES	ER (IC50 M)[5]	Percentage Change (%)	ERA (IC50 (µM))[6]	Docking Score	MM-GBSA	NR-toxpred	Computational prediction
DTXSID8031865	PFOA	1.82×10^{-3}	1.31×10^{7}	469.5 ± 4.6	-8.006	-33.2859	Antagonist	Inactive
DTXSID3031864	PFOS	2.01×10^{-4}	1.45×10^{6}	-NA-	-8.353	-45.0419	Inactive	Inactive
DTXSID8031861	PFDoA	6.51×10^{-4}	4.68×10^{6}	-NA-	-7.609	-45.7217	Antagonist	Weak Antagonist
DTXSID3031860	PFDA	2.34×10^{-4}	1.68×10^{6}	-NA-	-8.331	-41.4015	Antagonist	Inactive
DTXSID8047553	PFUnDA	1.01×10^{-3}	7.27×10^{6}	-NA-	-7.575	-44.588	Antagonist	Weak Antagonist
DTXSID90868151	PFTrDA	5.22×10^{-4}	3.76×10^{6}	-NA-	-8.021	-46.2472	Antagonist	Weak Antagonist
DTXSID3040148	PFDS	1.52×10^{-4}	1.09×10^{6}	-NA-	-7.619	-48.2025	Inactive	Inactive
DTXSID8059970	PFPA	2.89×10^{-3}	2.08×10^{7}	-NA-	-5.081	-16.1773	Inactive	Inactive
DTXSID3031862	PFHxA	1.22×10^{-3}	8.78×10^{6}	-NA-	-6.966	-27.0587	Inactive	Inactive
DTXSID1037303	PFHpA	1.78×10^{-3}	1.28×10^{6}	-NA-	-7.842	-31.0619	Antagonist	Inactive
DTXSID2044630	HFPO-TA	-NA-	-NA-	190.1 ± 2.2	-6.586	-36.026	Unreliable	Inactive
DTXSID40880242	HFPO-TeA	-NA-	-NA-	8.2 ± 0.4	-8.086	-43.0197	Unreliable	Inactive

*The value of Estradiol's IC50, which has been used as the reference for the percentage change calculations, is M 1.39×10^{-8} M

Table S4: Comparison of ERB Predictions with Experimental Data for Perfluoroalkyl Substances (PFASs)

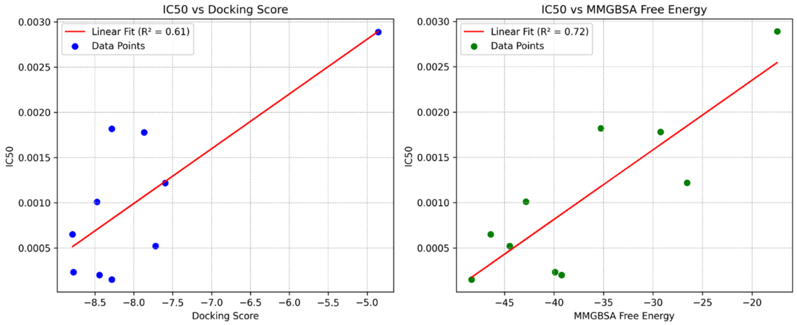

DTXSID	NAMES	ER (IC50 (µM)) [5]	Percentage Change (%)	ERB (IC50 (µM))[6]	Docking Score (kcal/mol)	MM-GBSA (kcal/mol)	NR-toxpred	Computational prediction
DTXSID8031865	PFOA	1.82×10^{-3}	1.31×10^{7}	384.4 ± 3.4	-8.283	-35.27	Inactive	Inactive
DTXSID3031864	PFOS	2.01×10^{-4}	1.45×10^{6}	-NA-	-8.442	-39.23	Inactive	Inactive
DTXSID8031861	PFDoA	6.51×10^{-4}	4.68×10^{6}	-NA-	-8.788	-46.40	Inactive	Inactive
DTXSID3031860	PFDA	2.34×10^{-4}	1.68×10^{6}	-NA-	-8.777	-39.90	Inactive	Inactive
DTXSID8047553	PFUnDA	1.01×10^{-3}	7.27×10^{6}	-NA-	-8.473	-42.83	Inactive	Inactive
DTXSID90868151	PFTrDA	5.22×10^{-4}	3.76×10^{6}	-NA-	-7.721	-44.46	Inactive	Inactive
DTXSID3040148	PFDS	1.52×10^{-4}	1.09×10^{6}	-NA-	-8.283	-48.33	Inactive	Inactive
DTXSID8059970	PFPA	2.89×10^{-3}	2.08×10^{7}	-NA-	-4.856	-17.46	Inactive	Inactive
DTXSID3031862	PFHxA	1.22×10^{-3}	8.78×10^{6}	-NA-	-7.597	-26.57	Inactive	Inactive
DTXSID1037303	PFHpA	1.78×10^{-3}	1.28×10^{6}	-NA-	-7.869	-29.25	Inactive	Inactive
DTXSID2044630	HFPO-TA	-NA-	-NA-	146.8 ± 1.7	-7.018	-36.86	Unreliable	Inactive
DTXSID40880242	HFPO-TeA	-NA-	-NA-	9.2 ± 0.6	-6.923	-37.08	Unreliable	Inactive

*The value of Estradiol's IC50, which has been used as the reference for the percentage change calculations, is M 1.39×10^{-8} M

Table S5: Comparison of PPARD Predictions with Experimental Data for Perfluoroalkyl Substances (PFASs)

DTXSID	NAMES	PPARD (IC50 (μM))[7]	Percentage Change (%)	Docking Score (kcal/mol)	MM-GBSA (kcal/mol)	NR-toxpred	Computational prediction
DTXSID3059921	PFTeDA	110.8 ± 10.5	+256.27	-10.494	-47.47	Inactive	Inactive
DTXSID8031863	PFNA	127.9 ± 7.0	+311.25	-8.864	-35.57	Inactive	Inactive
DTXSID3031862	PFHxA	159.6 ± 11.5	+413.18	-9.79	-26.23	Inactive	Inactive
DTXSID8031861	PFDoA	32.6 ± 6.1	+4.82	-9.235	-40.98	Inactive	Inactive
DTXSID8047553	PFUnDA	47.7 ± 6.3	+53.38	-10.766	-37.70	Inactive	Inactive
DTXSID90868151	PFTrDA	52.2 ± 5.8	+67.85	-11.607	-45.09	Inactive	Inactive
DTXSID3031860	PFDA	56.6 ± 14.3	+81.99	-11.209	-34.43	Inactive	Inactive
DTXSID3031864	PFOS	76.9 ± 9.2	+147.27	-7.687	-39.55	Inactive	Inactive

*The value of Linoleic acid IC50 (μM), which has been used as the reference for the percentage change calculations, is 31.1 μM

Table S6: Comparison of PPARA Predictions with Experimental Data for Perfluoroalkyl Substances (PFASs)

**DTXSID**	**NAMES**	**Potency ((EC50) uM)**[8]	**Percent Change form control (%)**	**Docking Score (kcal/mol)**	**MM-GBSA (kcal/mol)**	**Computational prediction**
DTXSID8031865	PFOA	9.5	950,000,000	-9.235	-31.47	Inactive
DTXSID3031864	PFOS	24	2,400,000,000	-9.196	-39.79	Inactive
DTXSID3031862	PFHxA	61	6,100,000,000	-9.726	-25.80	Inactive
DTXSID1037303	PFHpA	45	4,500,000,000	-10.438	-28.58	Inactive
DTXSID70880215	GenX	2.1	210,000,000	-9.085	-27.17	Inactive
DTXSID8031863	PFNA	9.6	960,000,000	-10.338	-30.61	Inactive

Positive control is GW7647 at 1×10^{-6}M.[8]

Table S7: Shortlisted commercially important PFAS for different nuclear receptors.

Receptor	DTXSID	NAMES	Docking Score (kcal/mol)	ΔG_{bind} (kcal/mol)	Primary Class	Secondary Class	Strength	Classification
**AR**								
	DTXSID7070925	N-ethyl-N-[2-(phosphonooxy)ethyl]perfluorooctanesulfonamide diammonium salt	-9.22	-60.24	Other aliphatics	PASF-based substances	Strong	Antagonist
	DTXSID9037743	2-(Perfluorodecyl)ethyl acrylate	-9.56	-53.12	Fluorotelomer PFAA precursors	n:2 FTACs	Moderate	Agonist
	DTXSID80865199	N-Methylperfluorooctanesulfonamidoethyl acrylate	-7.62	-59.78	FASA based PFAA precursors	N-Alkyl FASACs	Moderate	AGO-ANT
	DTXSID3069306	2-((Ethyl(pentadecafluoroheptyl)sulfonyl)amino)ethyl acrylate	-7.95	-56.52	FASA based PFAA precursors	N-Alkyl FASACs	Moderate	Antagonist
	DTXSID1071080	2-(Methyl((pentadecafluoroheptyl)sulfonyl)amino)ethyl acrylate	-9.17	-50.18	FASA based PFAA precursors	N-Alkyl FASACs	Moderate	AGO-ANT

	DTXSID5067348	2-(Perfluorooctyl)ethyl acrylate	-8.83	-44.34	Fluorotelomer PFAA precursors	n:2 FTACs	Weak	Agonist
	DTXSID10897307	Europium tri[3-(heptafluoropropylhydroxymethylene)]-(+)-camphorate	-8.63	-42.18	Other aliphatics, cyclic	Others, cyclic	Weak	Agonist
	DTXSID5059799	1H,1H-Perfluorooctyl acrylate	-8.71	-40.76	Other aliphatics	Ether based substances	Weak	AGO-ANT
	DTXSID7070507	2-(N-Methylperfluorobutylsulfonamido)ethyl acrylate	-7.48	-44.16	FASA based PFAA precursors	N-Alkyl FASACs	Weak	Agonist
**ERA**								
	DTXSID1070800	Perfluorohexadecanoic acid	-9.08	-47.54	PFAAs	PFCAs	Moderate	Antagonist
	DTXSID3059975	2-(N-Ethyl-N-(perfluorooctylsulfonyl)amino)ethyl acrylate	-8.30	-50.79	FASA based PFAA precursors	N-Alkyl FASACs	Moderate	AGO-ANT
	DTXSID6067836	1,1,2,2-Tetrahydroperfluorohexadecyl acrylate	-9.23	-46.02	Fluorotelomer PFAA precursors	n:2 FTACs	Moderate	Agonist
	DTXSID3059921	Perfluorotetradecanoic acid	-8.75	-46.96	PFAAs	PFCAs	Moderate	Antagonist

DTXSID80865199	N-Methylperfluorooctanesulfonamidoethyl acrylate	-7.89	-49.89	FASA based PFAA precursors	N-Alkyl FASACs	Weak	Antagonist
DTXSID1071080	2-(Methyl((pentadecafluoroheptyl)sulfonyl)amino)ethyl acrylate	-8.14	-48.70	FASA based PFAA precursors	N-Alkyl FASACs	Weak	Antagonist
DTXSID7070509	[N-Methylperfluorohexane-1-sulfonamide]ethyl acrylate	-8.36	-47.40	FASA based PFAA precursors	N-Alkyl FASACs	Weak	Antagonist
DTXSID90868151	Perfluorotridecanoic acid	-8.02	-46.25	PFAAs	PFCAs	Weak	Antagonist
DTXSID9037743	2-(Perfluorodecyl)ethyl acrylate	-8.13	-44.12	Fluorotelomer PFAA precursors	n:2 FTACs	Weak	Agonist
DTXSID8031861	Perfluorododecanoic acid	-7.61	-45.72	PFAAs	PFCAs	Weak	Antagonist
DTXSID5067348	2-(Perfluorooctyl)ethyl acrylate	-8.05	-42.48	Fluorotelomer PFAA precursors	n:2 FTACs	Weak	Agonist
DTXSID8047553	Perfluoroundecanoic acid	-7.58	-44.59	PFAAs	PFCAs	Weak	Antagonist

**PPARG**								
	DTXSID1066071	Perfluorooctadecanoic acid	-8.22	-49.96	PFAAs	PFCAs	Weak	Antagonist
**PPARA**								
	DTXSID1066071	Perfluorooctadecanoic acid	-14.22	-56.92	PFAAs	PFCAs	Strong	Active
	DTXSID1070800	Perfluorohexadecanoic acid	-12.40	-51.67	PFAAs	PFCAs	Strong	Active
	DTXSID50889140	1,3-Bis[4-(ethenyloxy)butyl] 2-(perfluorohexyl)ethylpropanedioate	-9.54	-70.22	Other aliphatics	Ether based substances	Strong	Active
	DTXSID3059921	Perfluorotetradecanoic acid	-12.82	-44.87	PFAAs	PFCAs	Strong	Active
	DTXSID4067513	Perfluoroeicosyl iodide	-9.42	-67.18	Perfluoro PFAA precursors	PFAIs	Strong	Active
	DTXSID60880406	2H-Tricosafluoro-5,8,11,14-tetrakis(trifluoromethyl)-3,6,9,12,15-pentaoxaoctadecane	-10.23	-60.97	Other aliphatics	Ether based substances	Strong	Active
	DTXSID4063660	(Perfluorotetradecyl)ethyl 2-methyl-2-propenoate	-10.46	-58.74	Fluorotelomer PFAA precursors	n:2 FTMACs	Strong	Active

DTXSID6067836	1,1,2,2-Tetrahydroperfluorohexadecyl acrylate	-9.22	-58.03	Fluorotelomer PFAA precursors	n:2 FTACs	Moderate	Active
DTXSID70880477	1,4-Bis((perfluorohexyl)ethyl) sulphonatosuccinate sodium	-7.14	-71.97	Other aliphatics	Others	Moderate	Active
DTXSID40861915	2-(N-Butylperfluorooctanesulfonamido)ethyl acrylate	-8.27	-63.67	FASA based PFAA precursors	N-Alkyl FASACs	Moderate	Active
DTXSID7070925	N-ethyl-N-[2-(phosphonooxy)ethyl]perfluorooctanesulfonamide diammonium salt	-9.95	-51.91	Other aliphatics	PASF-based substances	Moderate	Active
DTXSID4041284	6:2 Fluorotelomer sulfonamide betaine	-9.08	-57.40	Other aliphatics	PASF-based substances	Moderate	Active
DTXSID5067841	(Perfluorododecyl)ethyl 2-propenoate	-9.36	-53.54	Fluorotelomer PFAA precursors	n:2 FTACs	Moderate	Active
DTXSID4070322	2-(Perfluorotetradecyl)-1-iodoethane	-8.84	-54.78	Fluorotelomer PFAA precursors	n:2 FTIs	Weak	Active
DTXSID8059922	2-(N-Ethylperfluorooctanesulfonamido)ethyl methacrylate	-8.38	-57.89	FASA based PFAA precursors	N-Alkyl FASMACs	Weak	Active
DTXSID4069422	2-(Perfluorotetradecyl)ethanol	-9.49	-49.22	Fluorotelomer PFAA precursors	n:2 FTOHs	Weak	Active

DTXSID5059797	Nonacosafluoro-1-iodotetradecane	-9.53	-48.79	Perfluoro PFAA precursors	PFAIs	Weak	Active
DTXSID7062295	N-Butylheptadecafluoro-N-(2-hydroxyethyl)octanesulphonamide	-8.51	-54.91	FASA based PFAA precursors	(N-Alkyl) FASEs	Weak	Active
DTXSID2067535	1-Iodo-1H,1H,2H,2H-perfluorotetradecane	-9.26	-49.53	Fluorotelomer PFAA precursors	n:2 FTIs	Weak	Active
DTXSID80865199	N-Methylperfluorooctanesulfonamidoethyl acrylate	-8.28	-56.32	FASA based PFAA precursors	N-Alkyl FASACs	Weak	Active
DTXSID00880243	Fluoroether E4	-8.70	-53.02	Other aliphatics	Ether based substances	Weak	Active
DTXSID3059975	2-(N-Ethyl-N-(perfluorooctylsulfonyl)amino)ethyl acrylate	-8.24	-55.57	FASA based PFAA precursors	N-Alkyl FASACs	Weak	Active
DTXSID90868151	Perfluorotridecanoic acid	-9.81	-43.29	PFAAs	PFCAs	Weak	Active
DTXSID3071727	(Perfluorododecyl)ethylsulfonyl chloride	-8.37	-52.44	Other aliphatics	Others	Weak	Active
DTXSID6070221	2-(Perfluorohexadecyl)ethanol	-8.72	-49.90	Fluorotelomer PFAA precursors	n:2 FTOHs	Weak	Active
DTXSID10624392	2-(N-Methylperfluorooctanesulfonamido)acetic acid	-9.22	-46.18	FASA based PFAA precursors	(N-Alkyl) FASAAs	Weak	Active

DTXSID9037743	2-(Perfluorodecyl)ethyl acrylate	-8.89	-46.97	Fluorotelomer PFAA precursors	n:2 FTACs	Weak	Active
DTXSID6062204	10:2 Fluorotelomer methacrylate	-8.39	-49.17	Fluorotelomer PFAA precursors	n:2 FTMACs	Weak	Active
DTXSID1070513	Potassium N-ethyl-N-((pentadecafluoroheptyl)sulphonyl)glycinate	-9.09	-44.23	FASA based PFAA precursors	(N-Alkyl) FASAAs	Weak	Active
DTXSID8031861	Perfluorododecanoic acid	-9.33	-41.33	PFAAs	PFCAs	Weak	Active
DTXSID8062101	2-(Perfluorooctyl)ethyl methacrylate	-8.83	-44.61	Fluorotelomer PFAA precursors	n:2 FTMACs	Weak	Active
DTXSID3069306	2-((Ethyl(pentadecafluoroheptyl)sulfonyl)amino)ethyl acrylate	-7.68	-51.70	FASA based PFAA precursors	N-Alkyl FASACs	Weak	Active
DTXSID0059796	Pentacosafluoro-1-iodododecane	-8.86	-43.24	Perfluoro PFAA precursors	PFAIs	Weak	Active
DTXSID5062760	2-(N-Ethylperfluorooctanesulfonamido)acetic acid	-8.21	-47.26	FASA based PFAA precursors	(N-Alkyl) FASAAs	Weak	Active
DTXSID60880486	Potassium N-(perfluorooctylsulphonyl)-N-propylglycinate	-8.01	-48.65	FASA based PFAA precursors	(N-Alkyl) FASAAs	Weak	Active
DTXSID90881345	3-[(Perfluorooctane-1-sulfonyl)amino]-	-8.13	-47.83	Other aliphatics	PASF-based substances	Weak	Active

	N,N-dimethylpropan-1-amine N-oxide potassium						
DTXSID40892507	11-Chloroperfluoro-3-oxaundecanesulfonic acid	-8.22	-46.86	Other aliphatics	PASF-based substances	Weak	Active
DTXSID7070505	Potassium N-ethyl-N-(perfluorohexylsulphonyl)glycinate	-8.56	-43.07	FASA based PFAA precursors	(N-Alkyl) FASAAs	Weak	Active
DTXSID00192353	8:2 Fluorotelomer sulfonic acid	-8.74	-41.79	Fluorotelomer PFAA precursors	n:2 Fluorotelomer sulfonic acids	Weak	Active
DTXSID70881343	2-{Methyl[(nonafluorobutyl)sulfonyl]amino}ethyl phosphate	-8.22	-44.72	Other aliphatics	PASF-based substances	Weak	Active
DTXSID3068170	2-(Perfluorododecyl)ethanol	-8.20	-44.19	Fluorotelomer PFAA precursors	n:2 FTOHs	Weak	Active
DTXSID1071080	2-(Methyl((pentadecafluoroheptyl)sulfonyl)amino)ethyl acrylate	-7.37	-49.63	FASA based PFAA precursors	N-Alkyl FASACs	Weak	Active
DTXSID6070510	2-(N-Methylperfluorobutanesulfonamido)ethyl methacrylate	-7.64	-46.96	FASA based PFAA precursors	N-Alkyl FASMACs	Weak	Active
DTXSID10897307	Europium tri[3-(heptafluoropropylh	-8.26	-41.97	Other aliphatics, cyclic	Others, cyclic	Weak	Active

		ydroxymethylene)]-(+)-camphorate						
	DTXSID7027831	N-Methyl-N-(2-hydroxyethyl)perfluorooctanesulfonamide	-7.58	-46.62	FASA based PFAA precursors	(N-Alkyl) FASEs	Weak	Active
	DTXSID1067330	3,3,4,4,5,5,6,6,7,7,8,8,9,9,10,10,11,11,12,12,12-Henicosafluorododecane-1-sulphonyl chloride	-8.10	-42.63	Other aliphatics	Others	Weak	Active
	DTXSID5059878	Perfluorohexadecyl iodide	-7.68	-45.28	Perfluoro PFAA precursors	PFAIs	Weak	Active
	DTXSID6027426	N-Ethyl-N-(2-hydroxyethyl)perfluorooctane sulfonamide	-7.34	-46.82	FASA based PFAA precursors	(N-Alkyl) FASEs	Weak	Active
	DTXSID1071664	Tridecafluoro-N-(2-hydroxyethyl)-N-methyl-1-hexanesulfonamide	-7.76	-42.73	FASA based PFAA precursors	(N-Alkyl) FASEs	Weak	Active
	DTXSID1062124	10:2 Fluorotelomer iodide	-7.63	-41.65	Fluorotelomer PFAA precursors	n:2 FTIs	Weak	Active
	DTXSID80889133	Dimethyl 2-(3,3,4,4,5,5,6,6,7,7,8,8,8-tridecafluorooctyl)-1,3-propanedioate	-7.13	-42.28	Other aliphatics	Ether based substances	Weak	Active
**MR**								
	DTXSID5062760	2-(N-Ethylperfluorooctan	-11.12	-58.36	FASA based PFAA precursors	(N-Alkyl) FASAAs	Moderate	Active

	esulfonamido)acetic acid						
DTXSID6067836	1,1,2,2-Tetrahydroperfluorohexadecyl acrylate	-9.99	-61.62	Fluorotelomer PFAA precursors	n:2 FTACs	Moderate	Active
DTXSID9067514	Perfluorooctadecyl iodide	-10.33	-59.52	Perfluoro PFAA precursors	PFAIs	Moderate	Active
DTXSID7070925	N-ethyl-N-[2-(phosphonooxy)ethyl]perfluorooctanesulfonamide diammonium salt	-8.76	-63.33	Other aliphatics	PASF-based substances	Weak	Active
DTXSID4059835	Fluorocarbon FC 70	-9.47	-54.85	Other aliphatics	Others	Weak	Active
DTXSID3040148	Perfluorodecanesulfonic acid	-8.96	-56.25	PFAAs	PFSAs	Weak	Active
DTXSID7070509	[N-Methylperfluorohexane-1-sulfonamide]ethyl acrylate	-8.41	-56.96	FASA based PFAA precursors	N-Alkyl FASACs	Weak	Active
DTXSID3068170	2-(Perfluorododecyl)ethanol	-9.74	-47.52	Fluorotelomer PFAA precursors	n:2 FTOHs	Weak	Active
DTXSID8031861	Perfluorododecanoic acid	-8.91	-51.86	PFAAs	PFCAs	Weak	Active
DTXSID4069422	2-(Perfluorotetradecyl)ethanol	-8.76	-51.93	Fluorotelomer PFAA precursors	n:2 FTOHs	Weak	Active
DTXSID5059878	Perfluorohexadecyl iodide	-8.42	-52.55	Perfluoro PFAA precursors	PFAIs	Weak	Active
DTXSID4070322	2-(Perfluorotetradecyl)-1-iodoethane	-8.60	-51.54	Fluorotelomer PFAA precursors	n:2 FTIs	Weak	Active

DTXSID30880615	Trimethyl-3-(((pentadecafluoroheptyl)sulphonyl)amino)propylammonium chloride	-8.43	-51.71	Other aliphatics	PASF-based substances	Weak	Active
DTXSID6027426	N-Ethyl-N-(2-hydroxyethyl)perfluorooctane sulfonamide	-8.51	-50.26	FASA based PFAA precursors	(N-Alkyl) FASEs	Weak	Active

Table S8: Primary Class and Secondary Class in the shortlisted PFAS against AR.

Primary Class	Secondary Class	Count
FASA-based PFAA precursors	(N-Alkyl) FASAAs	1
	N-Alkyl FASACs	4
	N-Alkyl FASAs	1
Fluorotelomer PFAA precursors	n:2 FTACs	6
Other aliphatics	Ether based substances	12
	Others	6
	PACF-based substances	1
	PASF-based substances	14
Other aliphatics, cyclic	Others, cyclic	1
Side-chain aromatics	Others	87
	PACF-based substances	2
	PASF-based substances	11
Steroid-Backbone	Steroid-Backbone	3

Table S9: Primary Class and Secondary Class in the shortlisted PFAS against ERA.

Primary Class	Secondary Class	Count
FASA-based PFAA precursors	N-Alkyl FASACs	6
Fluorotelomer PFAA precursors	n:2 FTACs	7
Other aliphatics	Amide based substances	4
	Ether based substances	6
	Others	11
	PACF-based substances	18
	PASF-based substances	12
	Polyfluorinated amides	6
Other aliphatics, cyclic	Others, cyclic	4
	PASF-based substances, cyclic	2
PFAAs	PFCAs	10
Side-chain aromatics	Others	150
	PACF-based substances	5
	PASF-based substances	13
Steroid-Backbone	Steroid-Backbone	3

Table S10: Primary Class and Secondary Class in the shortlisted PFAS against ERB.

Primary Class	Secondary Class	Count
Fluorotelomer PFAA precursors	n:2 FTACs	3
Other aliphatics	Ether based substances	4
	PACF-based substances	1
	PASF-based substances	3
Side-chain aromatics	Others	41
	PASF-based substances	4
Steroid-Backbone	Steroid-Backbone	2

Table S11: Primary Class and Secondary Class in the shortlisted PFAS against GR.

Primary Class	Secondary Class	Count
Side-chain aromatics	Others	5
	PASF-based substances	1
Steroid-Backbone	Steroid-Backbone	3

Table S12: Primary Class and Secondary Class in the shortlisted PFAS against PR.

**Primary Class**	**Secondary Class**	**Count**
Side-chain aromatics	Others	1
	PASF-based substances	1

Table S13: Primary Class and Secondary Class in the shortlisted PFAS against PPARG.

Primary Class	Secondary Class	Count
Fluorotelomer PFAA precursors	SFAenes	6
	n:2 FTACs	1
Other aliphatics	Amide based substances	6
	FTI	1
	PACF-based substances	1
	PASF-based substances	1
Other aliphatics, cyclic	Others, cyclic	1
PFAAs	PFCAs	2
Side-chain aromatics	Others	82
	PACF-based substances	1
	PASF-based substances	9

Table S14: Primary Class and Secondary Class in the shortlisted PFAS against PPARD.

Primary Class	Secondary Class	Count
FASA based PFAA precursors	N-Alkyl FASACs	1
Fluorotelomer PFAA precursors	n:2 FTACs	1
Other aliphatics	Ether based substances	1
	Others	1
	PASF-based substances	1
Side-chain aromatics	Others	8

Table S15: Primary Class and Secondary Class in the shortlisted PFAS against PPARA.

Primary Class	Secondary Class	Count
FASA based PFAA precursors	(N-Alkyl) FASAAs	5
	(N-Alkyl) FASEs	9
	N-Alkyl FASACs	7
	N-Alkyl FASAs	2
	N-Alkyl FASMACs	8
Fluorotelomer PFAA precursors	SFAenes	22
	SFAs	77
	n:1 FTOHs	3
	n:2 FTACs	9
	n:2 FTIs	9
	n:2 FTMACs	9
	n:2 FTOHs	6
	n:2 FTOs	3
	n:2 Fluorotelomer sulfonic acids	3
	n:2 Polyfluoroalkyl phosphoric acid esters, diester	8
	n:2 Polyfluoroalkyl phosphoric acid esters, monoester	9
Non-PFAA perfluoroalkyls	PFAenes	1
	PFAs	6
Other aliphatics	Amide based substances	17
	Ether and Amide based substances	1
	Ether and Amide based substances	5
	Ether and PACF-based substances	14
	Ether based substances	125
	FT	47
	FTBr	6
	FTCA	5
	FTCl	1
	FTI	55
	FTIOH	4
	FTOH	5
	FTPO3	11
	FTS	1
	Iodo based substances	5
	Others	140

	PACF-based substances	40
	PACF-based substances	2
	PASF-based substances	91
	Polyfluoralkanes	2
	Polyfluorinated amidesÃ,Â	24
Other aliphatics, cyclic	Others, cyclic	50
	PACF-based substances, cyclic	1
	PASF-based substances, cyclic	20
PFAAs	PFCAs	10
	PFECAs	6
	PFESAs	1
	PFPiAs	1
	PFSAs	10
Perfluoro PFAA precursors	PAFs	3
	PFAIs	9
Side-chain aromatics	Others	387
	PACF-based substances	10
	PASF-based substances	66

Table S16: Primary Class and Secondary Class in the shortlisted PFAS against MR.

Primary Class	Secondary Class	Count
FASA based PFAA precursors	(N-Alkyl) FASAAs	2
	(N-Alkyl) FASEs	3
	N-Alkyl FASACs	2
	N-Alkyl FASMACs	3
Fluorotelomer PFAA precursors	SFAenes	13
	SFAs	27
	n:1 FTOHs	1
	n:2 FTACs	1
	n:2 FTIs	1
	n:2 FTOHs	3
	n:2 FTOs	1
	n:2 Polyfluoroalkyl phosphoric acid esters, diester	1
	n:2 Polyfluoroalkyl phosphoric acid esters, monoester	2
Non-PFAA perfluoroalkyls	PFAs	2
Non-PFAA perfluoroalkyls, cyclic	PFAs, cyclic	2
Other aliphatics	Amide based substances	8
	Ether based substances	33
	FT	22
	FTBr	4
	FTCA	5
	FTCl	1
	FTI	29
	FTOH	5
	FTPO3	6
	Iodo based substances	3
	Others	62
	PACF-based substances	14
	PASF-based substances	31
	Polyfluorinated amidesÃ,Â	12
Other aliphatics, cyclic	Others, cyclic	22
	PACF-based substances, cyclic	1
	PASF-based substances, cyclic	7
PFAAs	PFCAs	6
	PFPAs	1
	PFSAs	4
Perfluoro PFAA precursors	PFAIs	2
Side-chain aromatics	Others	236
	PACF-based substances	6
	PASF-based substances	33
Steroid-Backbone	Steroid-Backbone	3

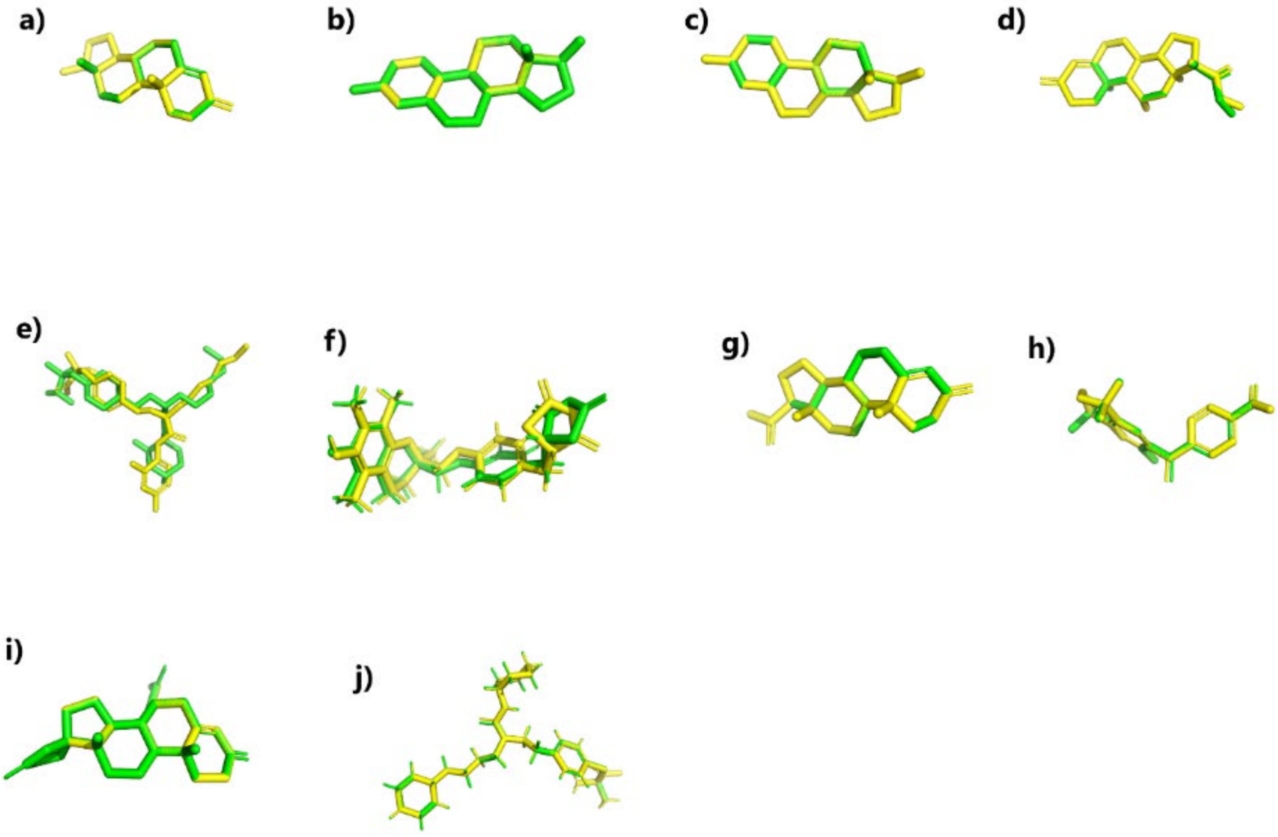

Figure S1: Superimposed structure of crystallographic ligand overlaid with its docked binding pose of a) AR, b) ERA, c) ERB, d) GR, e) PPARD, f) PPARG, g) PR, h) RXR, I) MR, j) PPARA ligands. Hydrogen atoms are not shown for clarity.

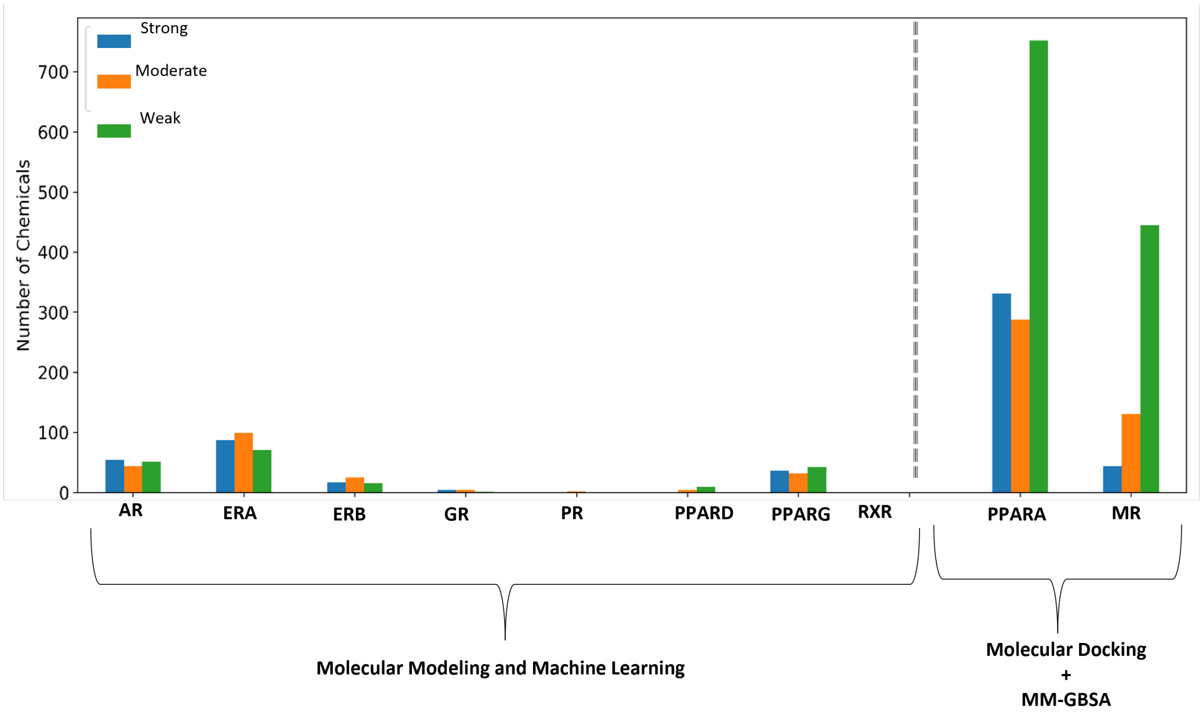

Figure S2: Distribution of PFASs by binding strength based on their average change in the docking score and ΔG_{bind} relative to reference ligands.

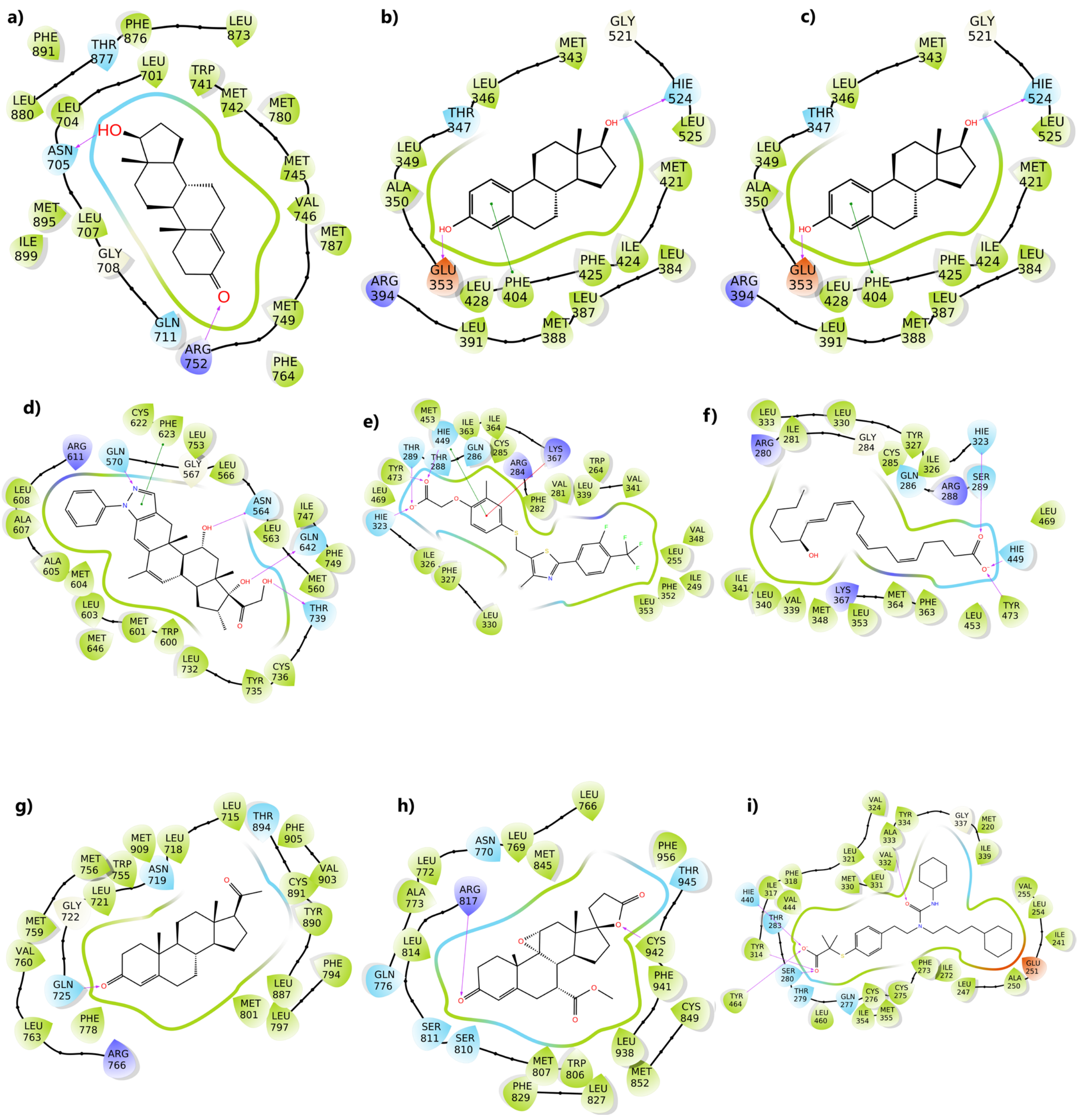

Figure S3: 2D interaction diagrams of a) AR -Testosterone b) ERA -17beta-Estradiol c) ERB - 17beta-Estradiol d) GR – deacylcortivazol, e) PPARD- GW0742 f) PPARG - 15-Hydroxyeicosatetraenoic acid g) PR - Progesterone, h) MR - Eplerenone and j) PPARA – GW-7647 complexes.

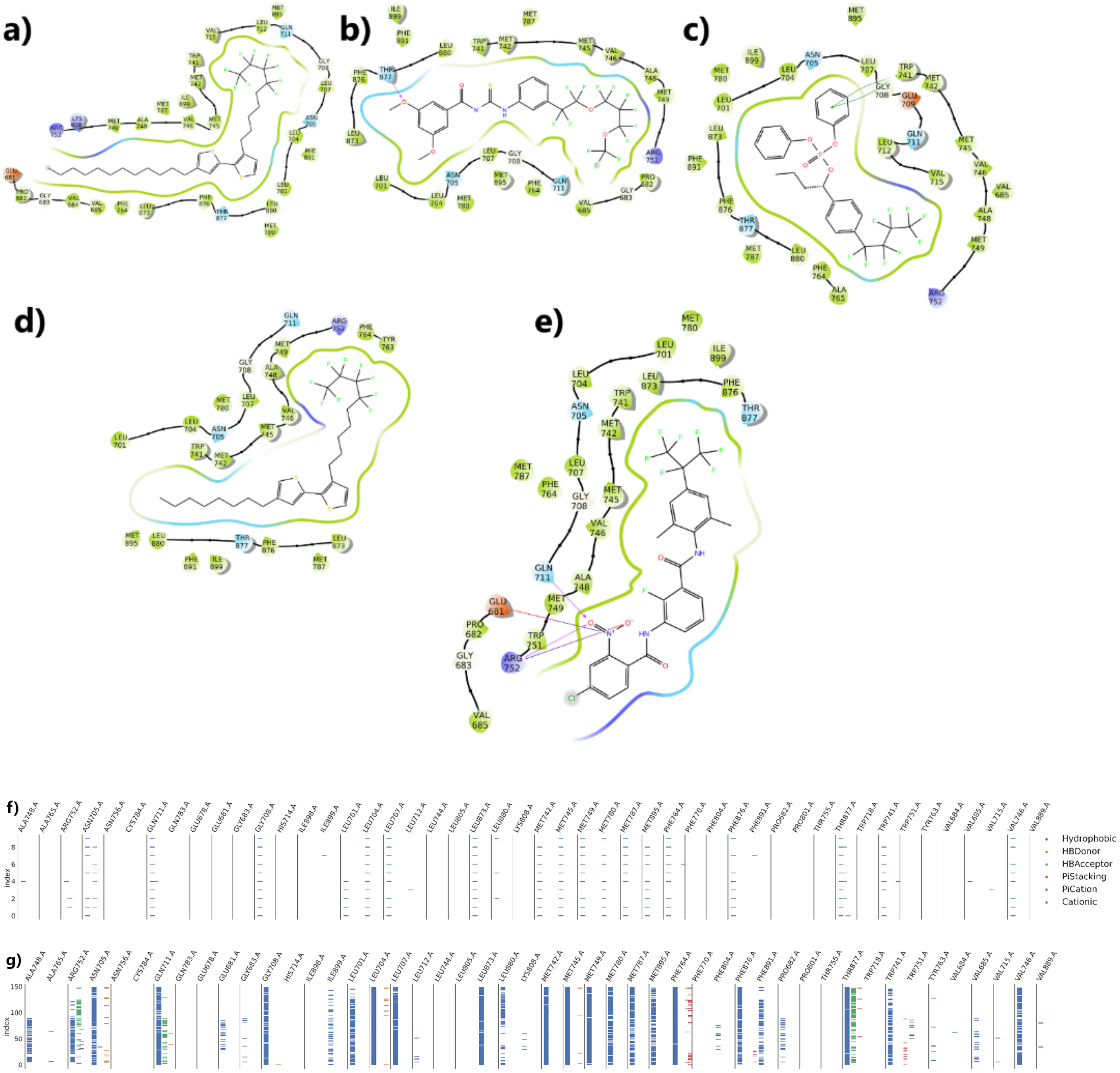

Figure S4: 2D interaction diagrams of top scoring PFAS chemicals a) DTXSID20844584 b) DTXSID10409808 c) DTXSID80827555 d) DTXSID80844585 and e) DTXSID40896469 with wild type AR. Interaction map of f) reference ligands and g) shortlisted PFAS chemicals against AR. Each column represents a residue, and each row corresponds to a ligand. The color of the markers indicates the type of interaction observed between the ligand and the residue.

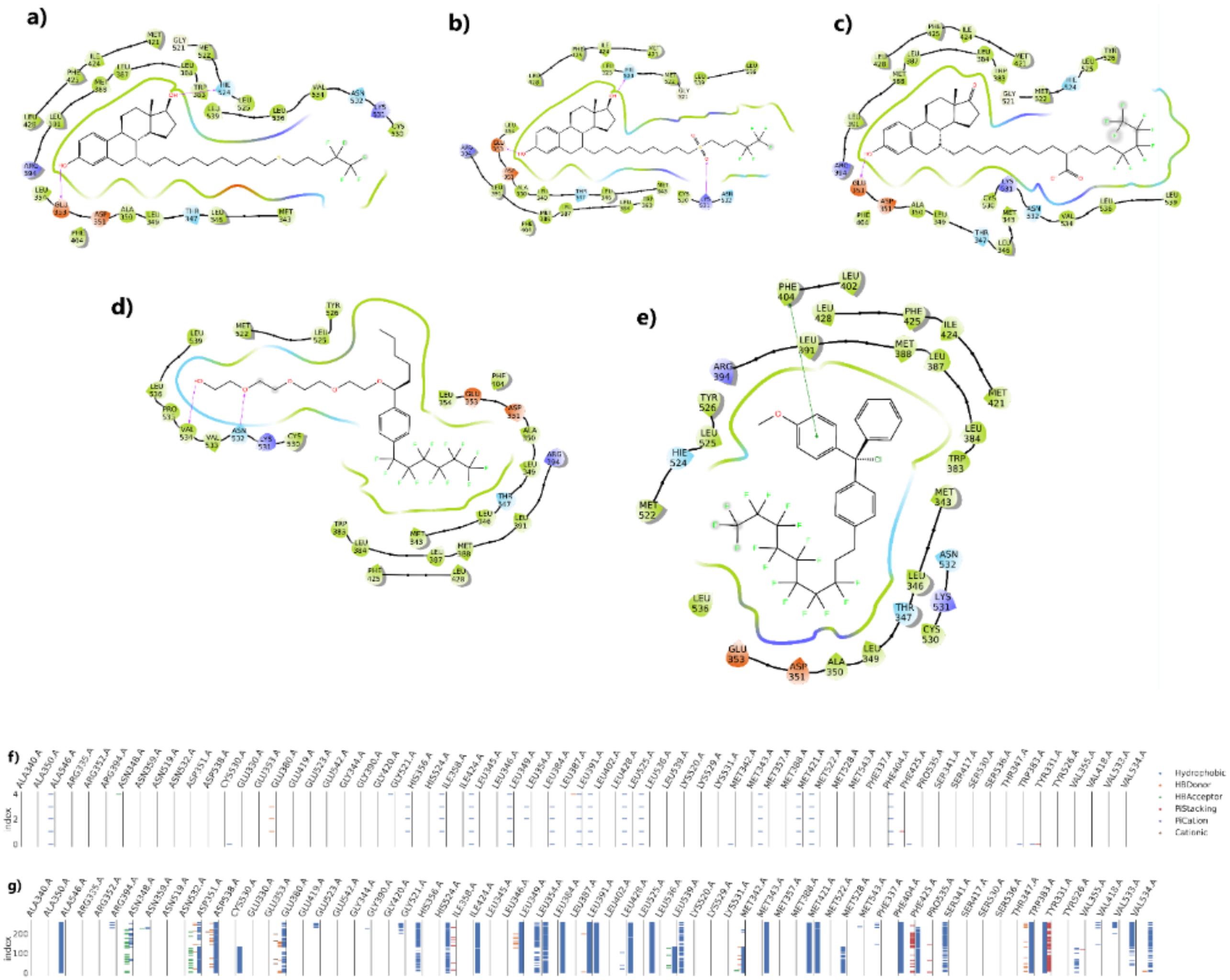

Figure S5: 2D interaction diagrams of top scoring PFAS chemicals a) DTXSID50462159 b) DTXSID90243368 c) DTXSID40897476 d) DTXSID90832413 and e) DTXSID00584835 with wild type ERA. Interaction map of f) known ligands and g) shortlisted PFAS chemicals against ERA. Each column represents a residue, and each row corresponds to a ligand. The color of the markers indicates the type of interaction observed between the ligand and the residue.

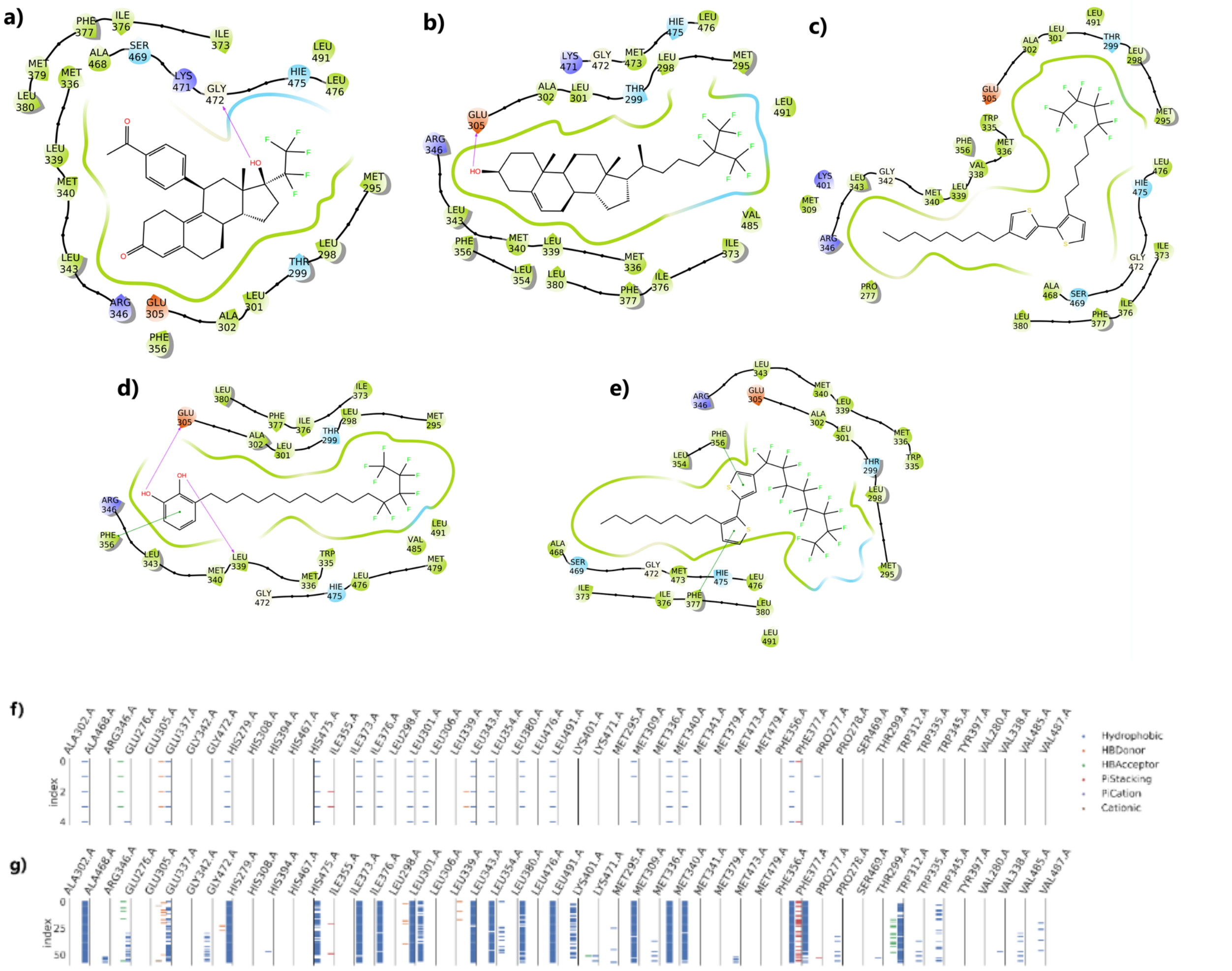

Figure S6: 2D interaction diagrams of top scoring PFAS chemicals with a) DTXSID60893551 b) DTXSID80897495 c) DTXSID80844585 d) DTXSID60895974 and e) DTXSID20827670 wild type ERB. Interaction map of f) known ligands and g) shortlisted PFAS chemicals against ERB. Each column represents a residue, and each row corresponds to a ligand. The color of the markers indicates the type of interaction observed between the ligand and the residue.

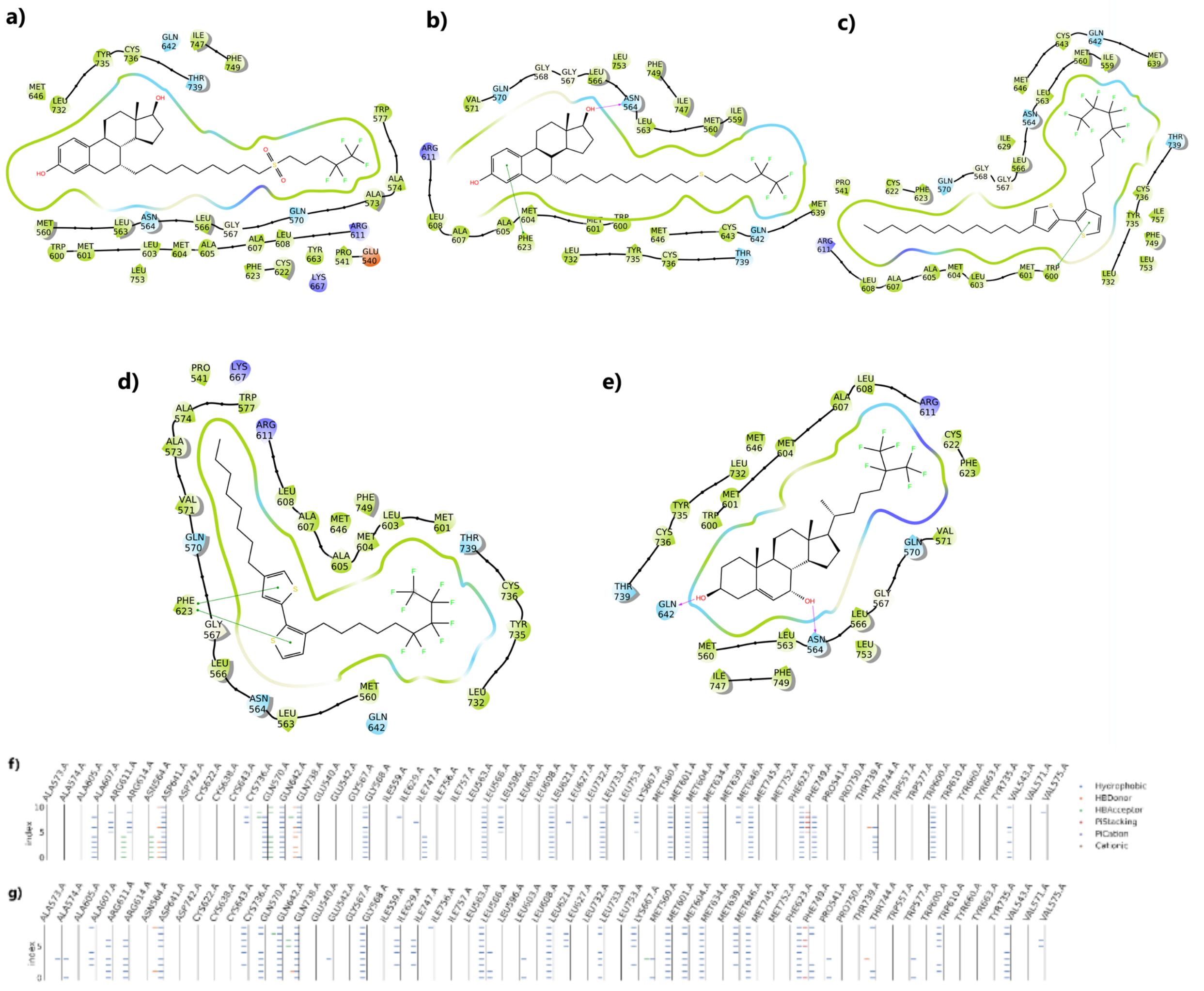

Figure S7: 2D interaction diagrams of top scoring PFAS chemicals a) DTXSID90243368 b) DTXSID50462159 c) DTXSID20844584 d) DTXSID80844585 and e) DTXSID40897496 with wild type GR. Interaction map of f) known ligands and g) shortlisted PFAS chemicals against GR. Each column represents a residue, and each row corresponds to a ligand. The color of the markers indicates the type of interaction observed between the ligand and the residue.

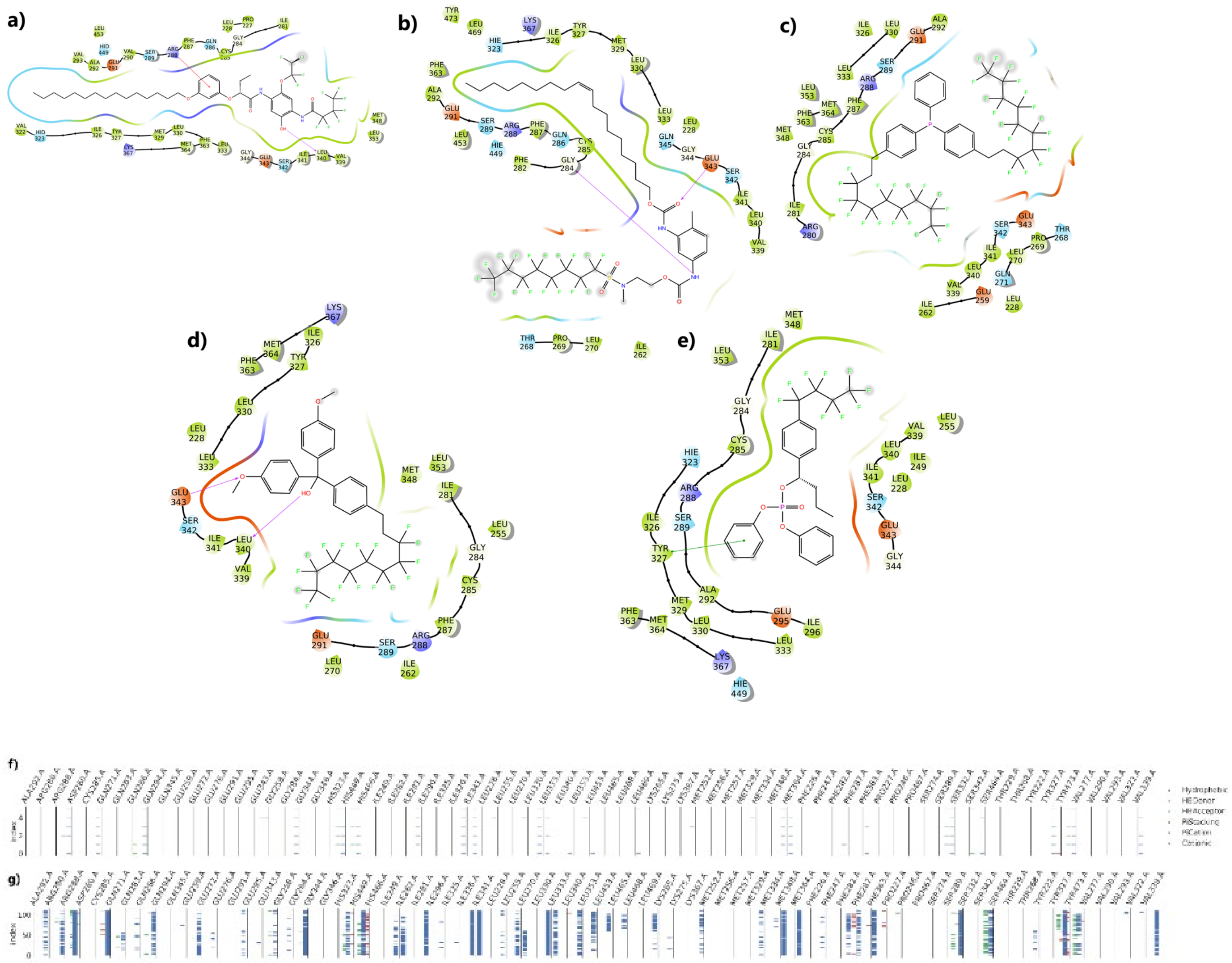

Figure S8: 2D interaction diagrams of top scoring PFAS chemicals a) DTXSID00896288 b) DTXSID60881195 c) DTXSID60475159 d) DTXSID10584841 and e) DTXSID80827555 with wild type PPARG. Interaction map of f) known ligands and g) shortlisted PFAS chemicals against PPARG. Each column represents a residue, and each row corresponds to a ligand. The color of the markers indicates the type of interaction observed between the ligand and the residue.

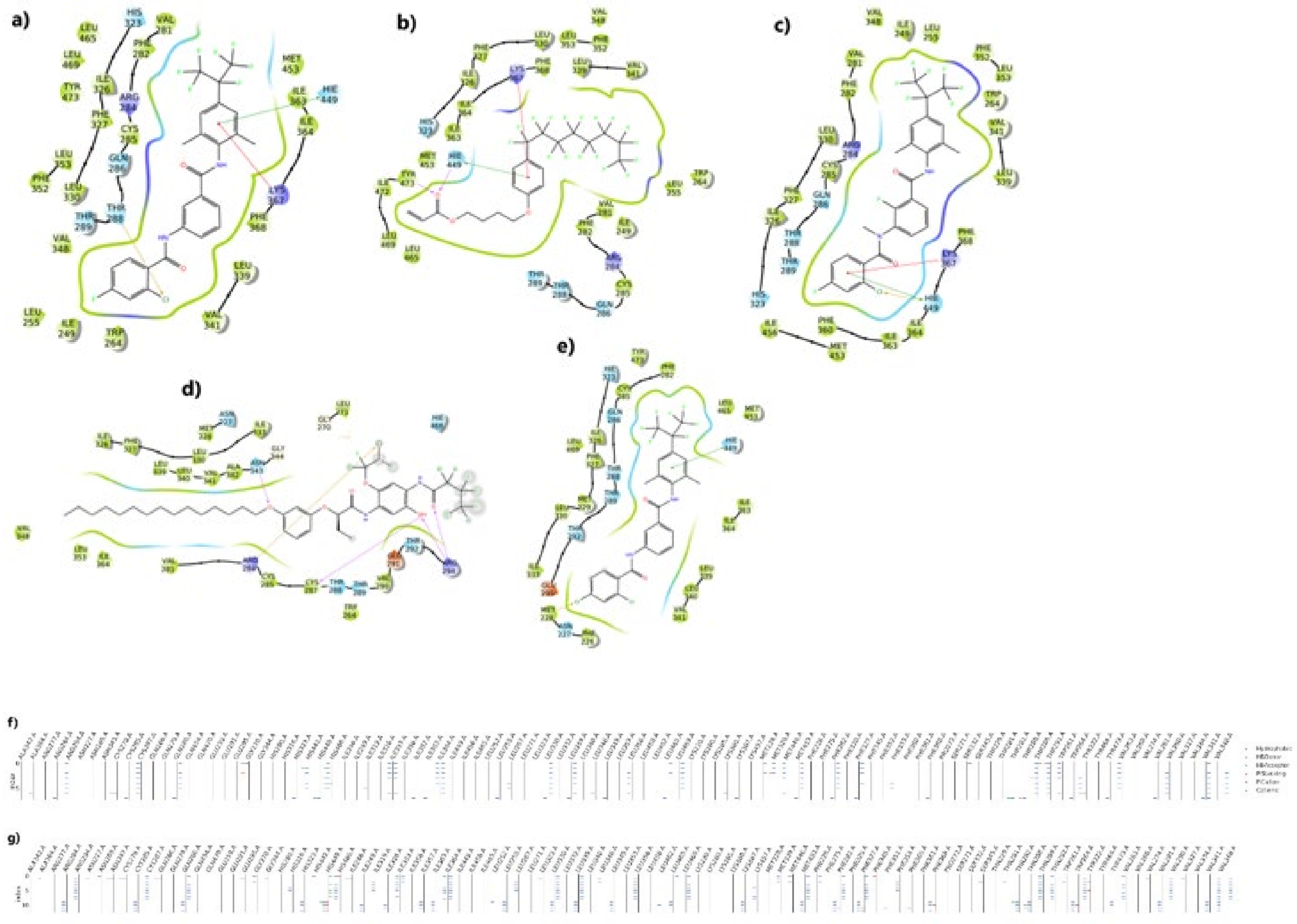

Figure S9: 2D interaction diagrams of top scoring a) DTXSID30896453 b) DTXSID60800381, c) DTXSID20896467, d) DTXSID00896288 and e) DTXSID10896456 PFAS chemicals with wild type PPARD. Interaction map of f) known ligands and g) shortlisted PFAS chemicals against PPARD. Each column represents a residue, and each row corresponds to a ligand. The color of the markers indicates the type of interaction observed between the ligand and the residue.

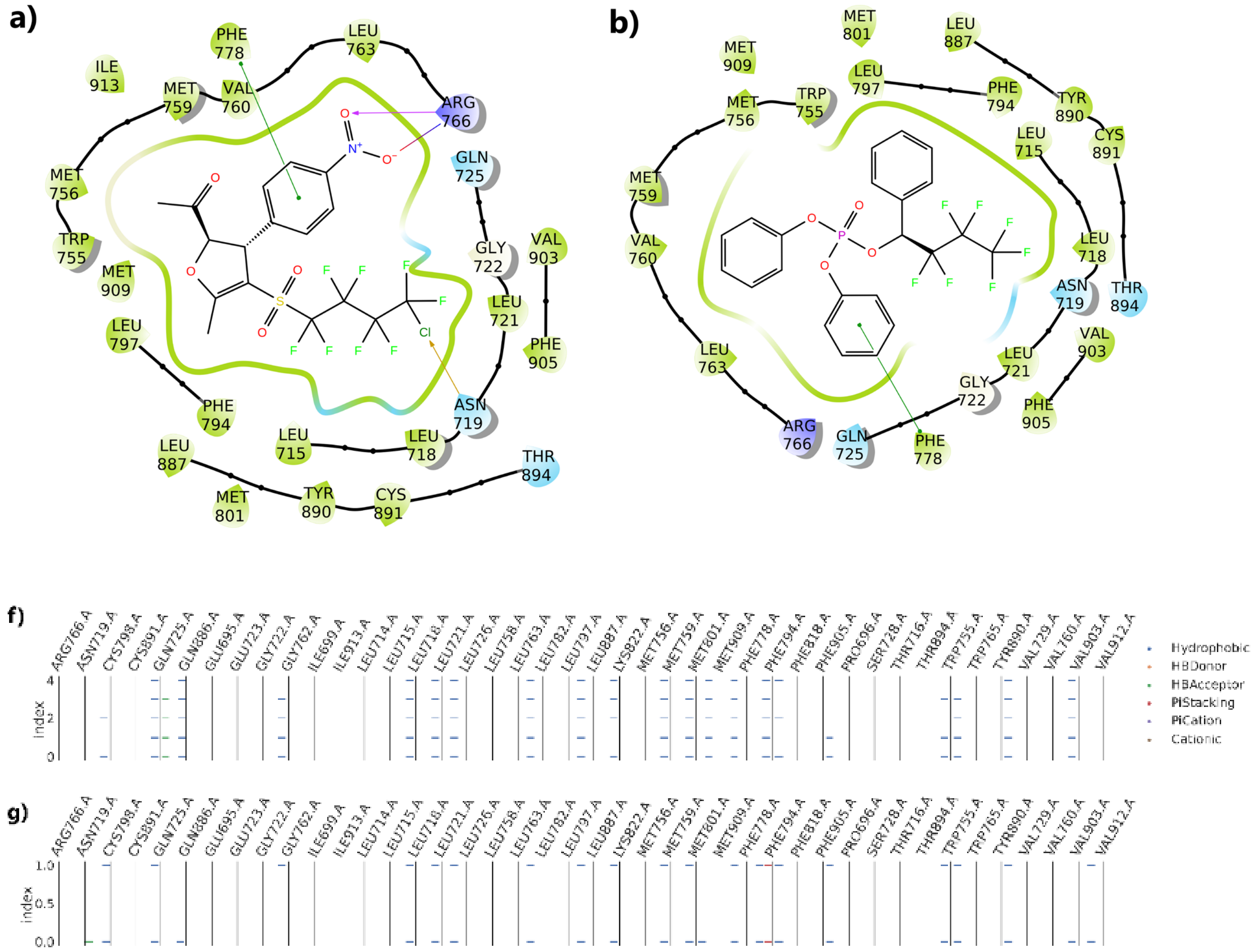

Figure S10: 2D interaction diagrams of top scoring a) DTXSID50702567 and b) DTXSID80896125 PFAS chemicals with wild type PR. Interaction map of f) known ligands and g) shortlisted PFAS chemicals against PR. Each column represents a residue, and each row corresponds to a ligand. The color of the markers indicates the type of interaction observed between the ligand and the residue.

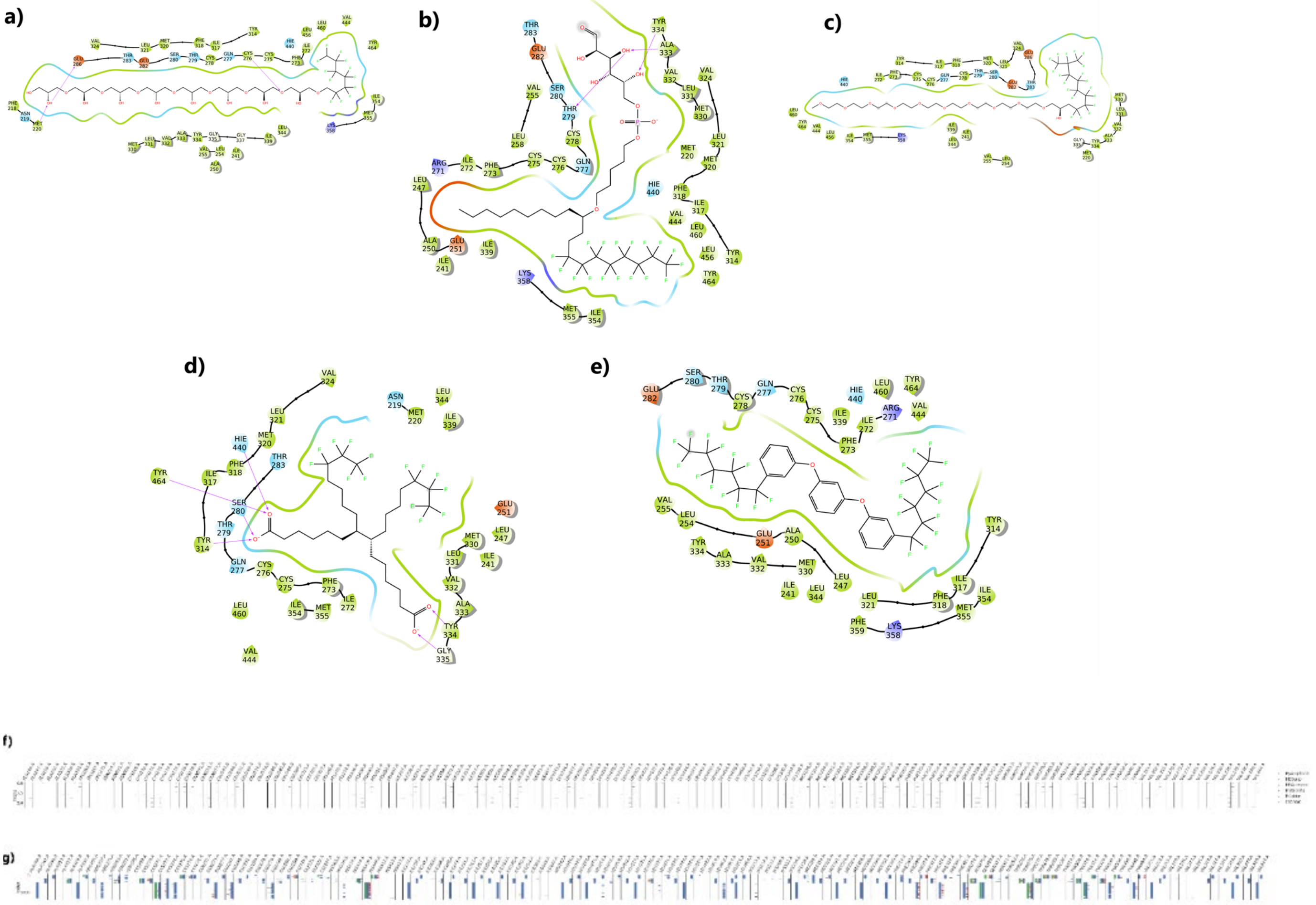

Figure S11: 2D interaction diagrams of top scoring a) DTXSID10810516, b) DTXSID20897313, c) DTXSID50881028, d) DTXSID00791668 and e) DTXSID60896168 PFAS chemicals with wild type PPARA

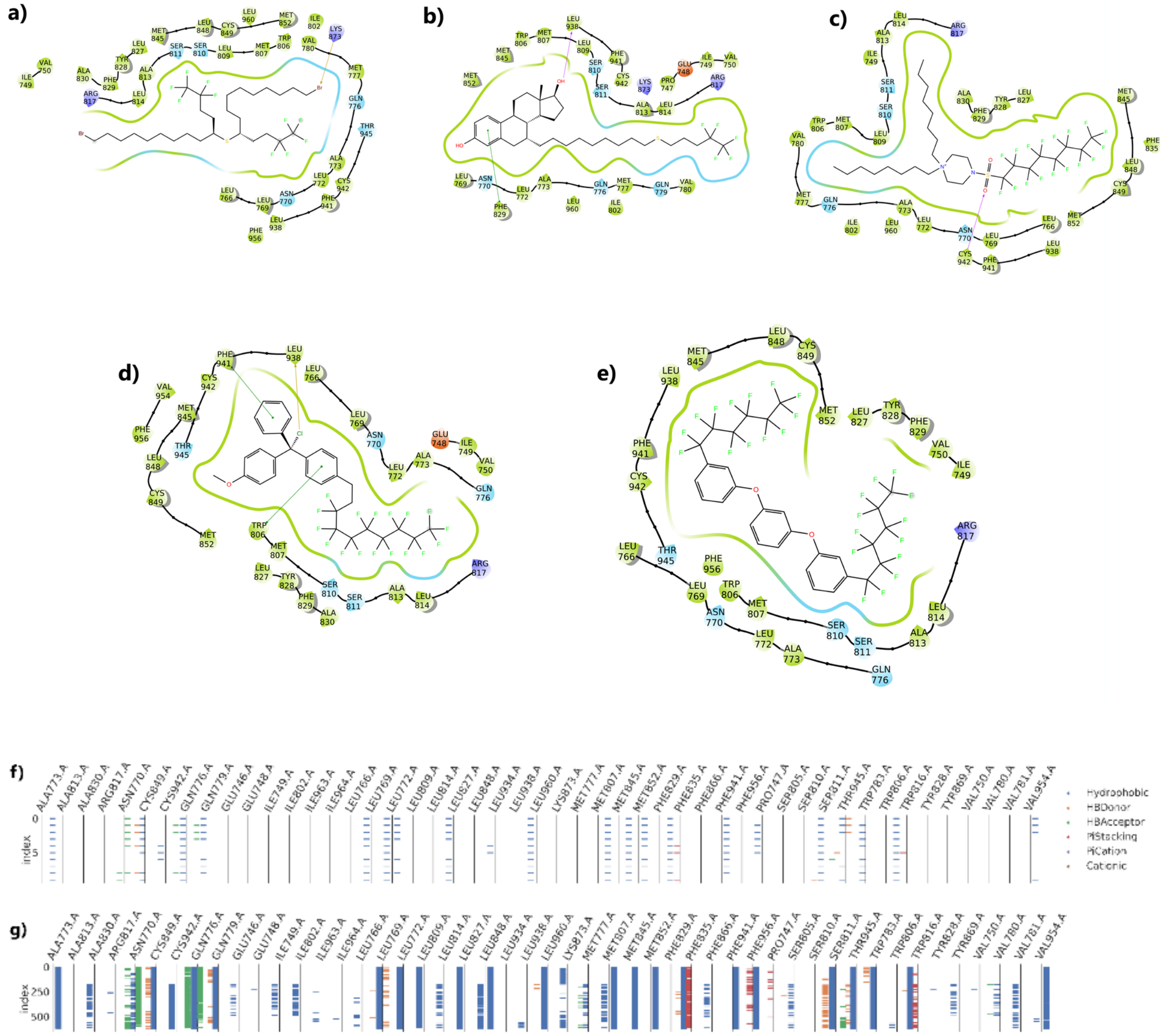

Figure S12: 2D interaction diagrams of top scoring a) DTXSID40692928 b) DTXSID50462159 c) DTXSID30897485, d) DTXSID00584835 and e) DTXSID60896168 PFAS chemicals with wild type M. Interaction map of f) known ligands and g) shortlisted PFAS chemicals against MR. Each column represents a residue, and each row corresponds to a ligand. The color of the markers indicates the type of interaction observed between the ligand and the residue.

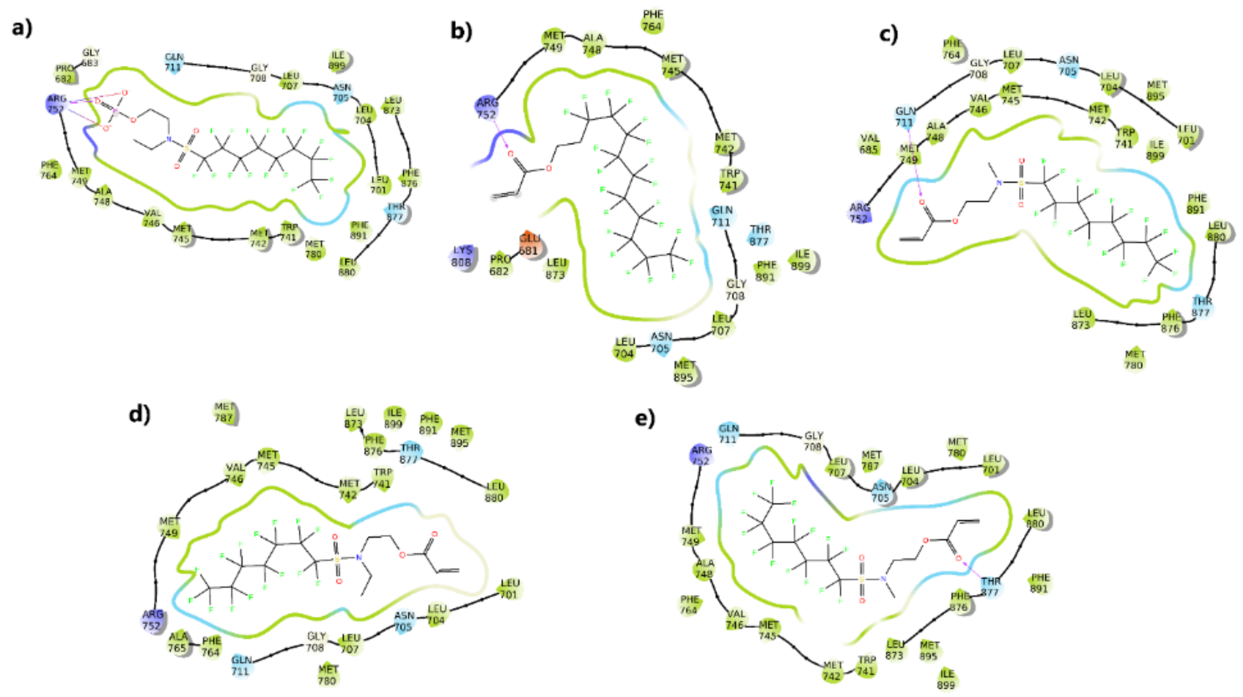

Figure S13: 2D interaction diagrams of top scoring commercially important PFAS chemicals a) DTXSID7070925, b) DTXSID9037743, c) DTXSID80865199, d) DTXSID3069306 and e) DTXSID1071080 with wild type AR.

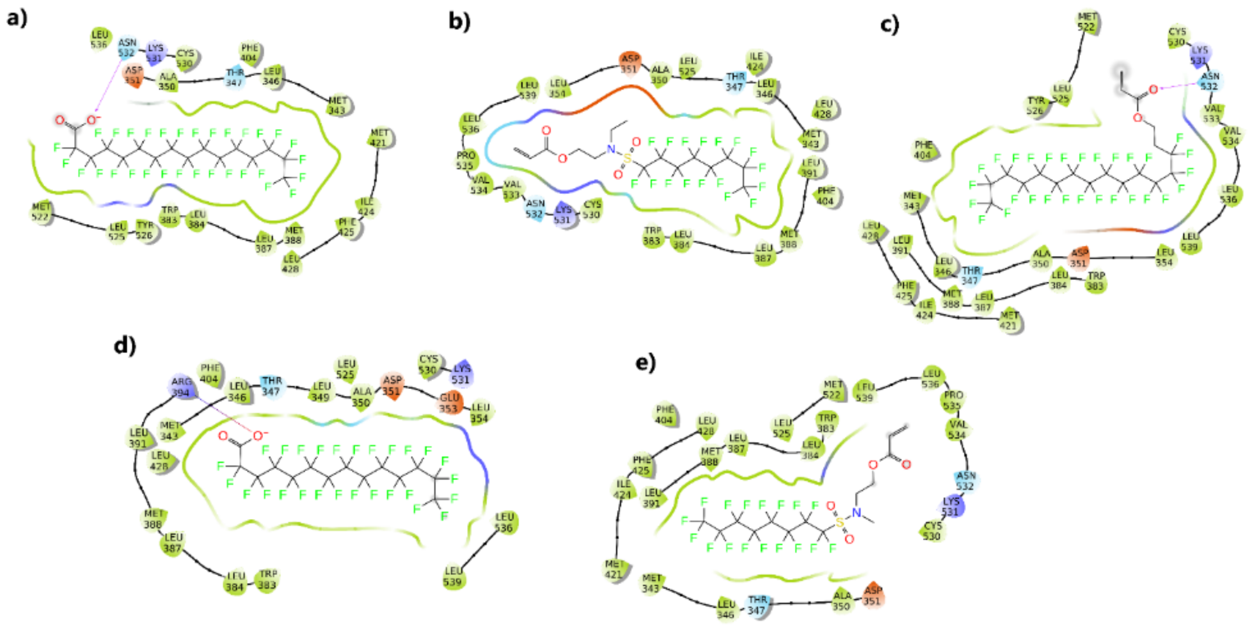

Figure S14: 2D interaction diagrams of top scoring commercially important PFAS chemicals a) DTXSID1070800, b) DTXSID3059975, c) DTXSID6067836, d) DTXSID3059921, and e) DTXSID80865199 with wild type ERA.

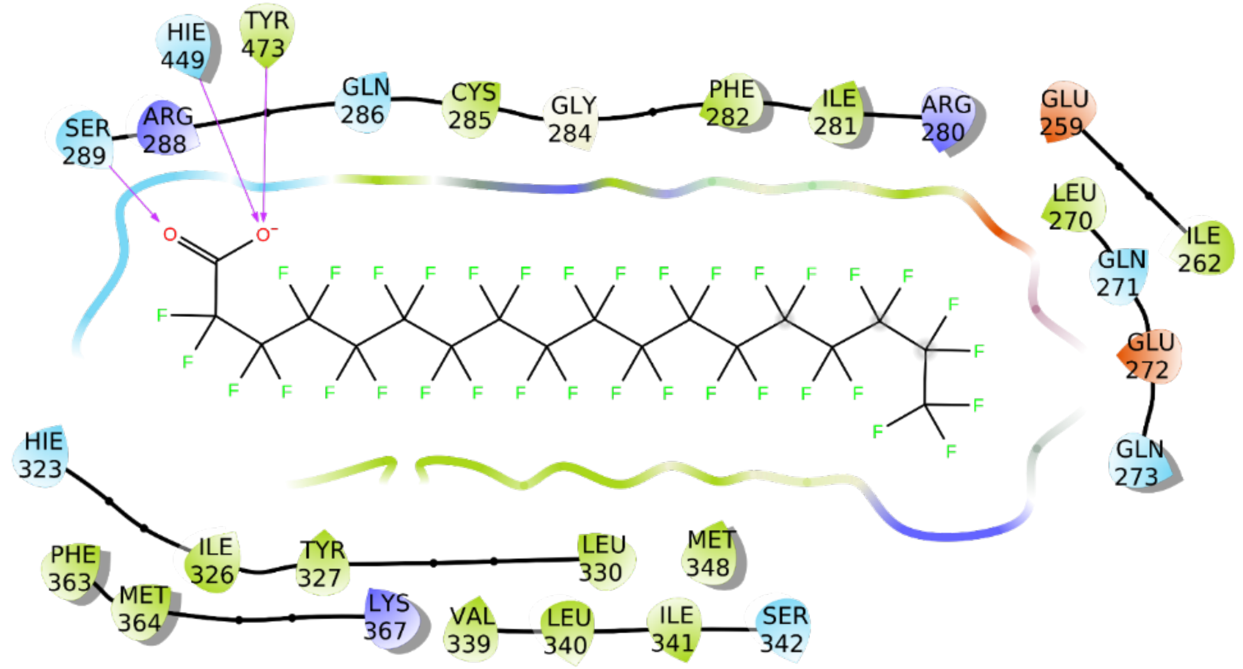

Figure S15: 2D interaction diagrams of top scoring commercially important PFAS chemicals a) DTXSID1066071 with wild type PPARG.

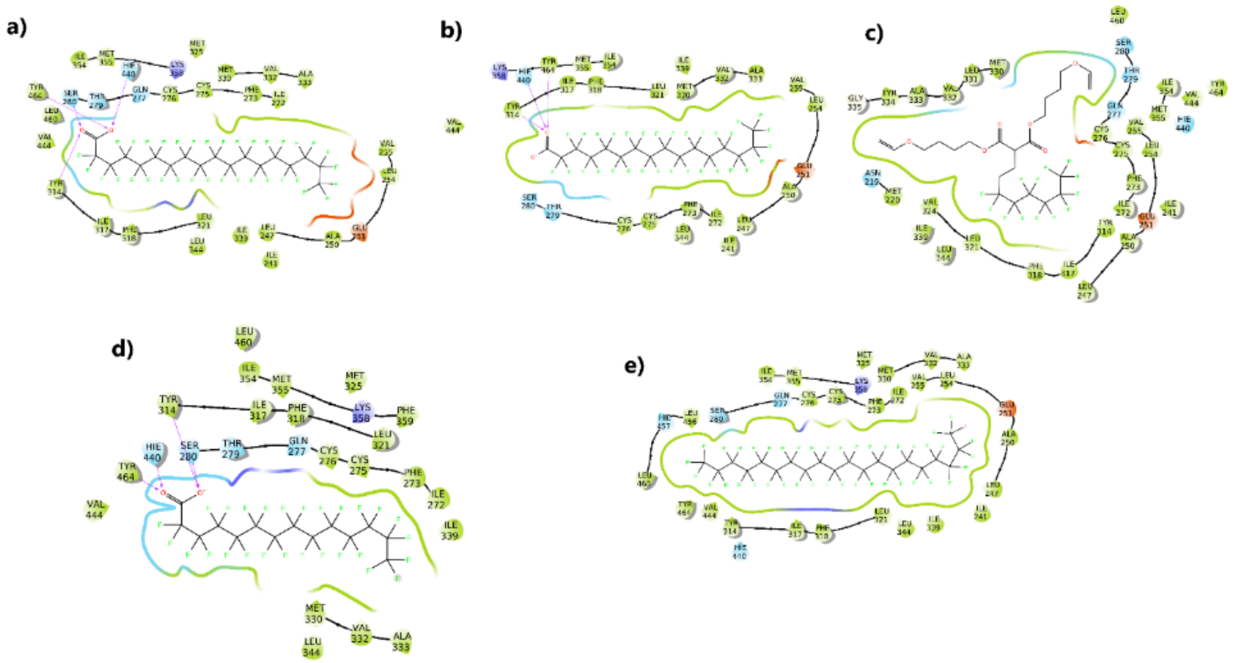

Figure S16: 2D interaction diagrams of top scoring commercially important PFAS chemicals a) DTXSID1066071 b) DTXSID1070800, c) DTXSID50889140, d) DTXSID3059921 and e) DTXSID4067513 with wild type PPARA.

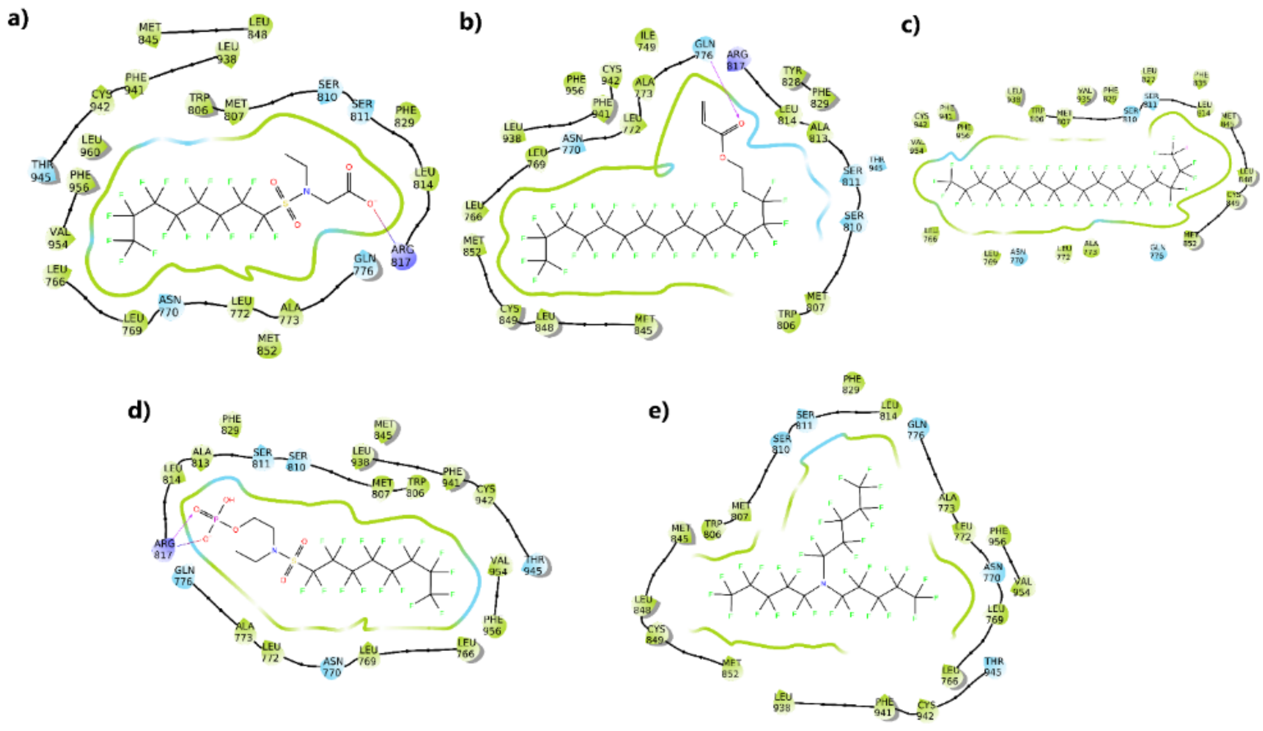

Figure S17: 2D interaction diagrams of top scoring commercially important PFAS chemicals a) DTXSID5062760, b) DTXSID6067836, c) DTXSID9067514, d) DTXSID7070925 and e) DTXSID4059835 with wild type MR.

References

(1) Srinivasan, J.; Miller, J.; Kollman, P. A.; Case, D. A. Continuum Solvent Studies of the Stability of RNA Hairpin Loops and Helices. *J. Biomol. Struct. Dyn.* **1998**, *16* (3), 671–682.

(2) Kollman, P. A.; Massova, I.; Reyes, C.; Kuhn, B.; Huo, S.; Chong, L.; Lee, M.; Lee, T.; Duan, Y.; Wang, W.; Donini, O.; Cieplak, P.; Srinivasan, J.; Case, D. A.; Cheatham, T. E., 3rd. Calculating Structures and Free Energies of Complex Molecules: Combining Molecular Mechanics and Continuum Models. *Acc. Chem. Res.* **2000**, *33* (12), 889–897.

(3) Case, D. A.; Metin Aktulga, H.; Belfon, K.; Ben-Shalom, I.; Brozell, S. R.; Cerutti, D. S.; Cheatham, T. E., III; Cruzeiro, V. W. D.; Darden, T. A.; Duke, R. E.; Giambasu, G.; Gilson, M. K.; Gohlke, H.; Goetz, A. W.; Harris, R.; Izadi, S.; Izmailov, S. A.; Jin, C.; Kasavajhala, K.; Kaymak, M. C.; King, E.; Kovalenko, A.; Kurtzman, T.; Lee, T.; LeGrand, S.; Li, P.; Lin, C.; Liu, J.; Luchko, T.; Luo, R.; Machado, M.; Man, V.; Manathunga, M.; Merz, K. M.; Miao, Y.; Mikhailovskii, O.; Monard, G.; Nguyen, H.; O'Hearn, K. A.; Onufriev, A.; Pan, F.; Pantano, S.; Qi, R.; Rahnamoun, A.; Roe, D. R.; Roitberg, A.; Sagui, C.; Schott-Verdugo, S.; Shen, J.; Simmerling, C. L.; Skrynnikov, N. R.; Smith, J.; Swails, J.; Walker, R. C.; Wang, J.; Wei, H.; Wolf, R. M.; Wu, X.; Xue, Y.; York, D. M.; Zhao, S.; Kollman, P. A. *Amber 2021*; University of California, San Francisco, 2021.

(4) Jakalian, A.; Bush, B. L.; Jack, D. B.; Bayly, C. I. Fast, Efficient Generation of High-Quality Atomic Charges. AM1-BCC Model: I. Method. *J. Comput. Chem.* **2000**, *21* (2), 132.

(5) Benninghoff, A. D.; Bisson, W. H.; Koch, D. C.; Ehresman, D. J.; Kolluri, S. K.; Williams, D. E. Estrogen-like Activity of Perfluoroalkyl Acids in Vivo and Interaction with Human and Rainbow Trout Estrogen Receptors in Vitro. *Toxicol. Sci.* **2011**, *120* (1), 42–58.

(6) Xin, Y.; Ren, X.-M.; Wan, B.; Guo, L.-H. Comparative in Vitro and in Vivo Evaluation of the Estrogenic Effect of Hexafluoropropylene Oxide Homologues. *Environ. Sci. Technol.* **2019**, *53* (14), 8371–8380.

(7) Li, C.-H.; Ren, X.-M.; Cao, L.-Y.; Qin, W.-P.; Guo, L.-H. Investigation of Binding and Activity of Perfluoroalkyl Substances to the Human Peroxisome Proliferator-Activated Receptor β/δ. *Environ. Sci. Process. Impacts* **2019**, *21* (11), 1908–1914.

(8) Nielsen, G.; Heiger-Bernays, W. J.; Schlezinger, J. J.; Webster, T. F. Predicting the Effects of Per- and Polyfluoroalkyl Substance Mixtures on Peroxisome Proliferator-Activated Receptor Alpha Activity in Vitro. *Toxicology* **2022**, *465*, 153024.
